# Hello darkness, my old friend: 3-KETOACYL-COENZYME A SYNTHASE4 is a branch point in the regulation of triacylglycerol synthesis in *Arabidopsis thaliana*

**DOI:** 10.1093/plcell/koad059

**Published:** 2023-03-03

**Authors:** Urszula Luzarowska, Anne-Kathrin Ruß, Jérôme Joubès, Marguerite Batsale, Jędrzej Szymański, Venkatesh P. Thirumalaikumar, Marcin Luzarowski, Si Wu, Feng Zhu, Niklas Endres, Sarah Khedhayir, Julia Schumacher, Weronika Jasinska, Ke Xu, Sandra Marcela Correa Cordoba, Simy Weil, Aleksandra Skirycz, Alisdair Robert Fernie, Yonghua Li-Beisson, Corina M Fusari, Yariv Brotman

**Affiliations:** Department of Life Sciences, Ben Gurion University of the Negev, 8410501 Beer-Sheva, Israel; Max Planck Institute of Molecular Plant Physiology, Am Mühlenberg 1, 14476 Potsdam-Golm, Germany; Laboratoire de Biogenèse Membranaire, UMR 5200, CNRS, University Bordeaux, F-33140 Villenave d’Ornon, France; Laboratoire de Biogenèse Membranaire, UMR 5200, CNRS, University Bordeaux, F-33140 Villenave d’Ornon, France; Department of Molecular Genetics, Leibniz Institute of Plant Genetics and Crop Plant Research, OT Gatersleben, 06466 Seeland, Germany; IBG-4 Bioinformatics, Forschungszentrum Jülich, 52428 Jülich, Germany; Max Planck Institute of Molecular Plant Physiology, Am Mühlenberg 1, 14476 Potsdam-Golm, Germany; Max Planck Institute of Molecular Plant Physiology, Am Mühlenberg 1, 14476 Potsdam-Golm, Germany; Department of Genetics, Stanford University School of Medicine, Stanford, CA 94305, USA; Max Planck Institute of Molecular Plant Physiology, Am Mühlenberg 1, 14476 Potsdam-Golm, Germany; National R&D Center for Citrus Preservation, Key Laboratory of Horticultural Plant Biology, Ministry of Education, Huazhong Agricultural University, Wuhan 430070, P.R. China; Max Planck Institute of Molecular Plant Physiology, Am Mühlenberg 1, 14476 Potsdam-Golm, Germany; Max Planck Institute of Molecular Plant Physiology, Am Mühlenberg 1, 14476 Potsdam-Golm, Germany; Department for Plant Cell and Molecular Biology, Institute for Biology, Humboldt-Universität zu Berlin, Philippstraße 13, 10115 Berlin, Germany; Department of Life Sciences, Ben Gurion University of the Negev, 8410501 Beer-Sheva, Israel; Max Planck Institute of Molecular Plant Physiology, Am Mühlenberg 1, 14476 Potsdam-Golm, Germany; Department of Life Sciences, Ben Gurion University of the Negev, 8410501 Beer-Sheva, Israel; Department of Life Sciences, Ben Gurion University of the Negev, 8410501 Beer-Sheva, Israel; Max Planck Institute of Molecular Plant Physiology, Am Mühlenberg 1, 14476 Potsdam-Golm, Germany; Max Planck Institute of Molecular Plant Physiology, Am Mühlenberg 1, 14476 Potsdam-Golm, Germany; CEA, CNRS, BIAM, Institute de Biosciences et Biotechnologies Aix-Marseille, Aix Marseille Univ., F-13108 Saint Paul-Lez-Durance, France; Centro de Estudios Fotosintéticos y Bioquímicos (CEFOBI-CONICET-UNR), Suipacha 570, S2000LRJ Rosario, Argentina; Department of Life Sciences, Ben Gurion University of the Negev, 8410501 Beer-Sheva, Israel

## Abstract

Plant lipids are important as alternative sources of carbon and energy when sugars or starch are limited. Here, we applied combined heat and darkness or extended darkness to a panel of ∼300 Arabidopsis (*Arabidopsis thaliana*) accessions to study lipid remodeling under carbon starvation. Natural allelic variation at *3-KETOACYL-COENZYME A SYNTHASE4* (*KCS4*), a gene encoding an enzyme involved in very long chain fatty acid (VLCFA) synthesis, underlies the differential accumulation of polyunsaturated triacylglycerols (puTAGs) under stress. Ectopic expression of *KCS4* in yeast and plants proved that KCS4 is a functional enzyme localized in the endoplasmic reticulum with specificity for C22 and C24 saturated acyl-CoA. Allelic mutants and transient overexpression in planta revealed the differential role of *KCS4* alleles in VLCFA synthesis and leaf wax coverage, puTAG accumulation, and biomass. Moreover, the region harboring *KCS4* is under high selective pressure and allelic variation at *KCS4* correlates with environmental parameters from the locales of Arabidopsis accessions. Our results provide evidence that KCS4 plays a decisive role in the subsequent fate of fatty acids released from chloroplast membrane lipids under carbon starvation. This work sheds light on both plant response mechanisms and the evolutionary events shaping the lipidome under carbon starvation.

## Teaser

Natural variation at *KCS4* underlies a differential accumulation of polyunsaturated triacylglycerols under carbon starvation, by acting as a branch point in the fate of fatty acids.

**IN A NUTSHELL**

**Background:** In the light, plants use photosynthesis to produce sugars and grow, while in the dark, they mainly use starch as a source of energy for maintenance throughout the night. When dark conditions last for a long time, plants consume all the available starch and enter a starvation mode, where they start feeding on lipids to support their metabolism. Although metabolism affects many responses, its genetic regulation is still underexplored. The study of complex traits such as metabolism can be facilitated by combining the use of natural populations and metabolomics. This strategy, called metabolomic-GWAS (genome-wide association studies), enables the identification of genes involved in the variation of multiple metabolic features under diverse environmental set-ups.

**Question:** How is metabolism controlled upon stress? What are the genes acting behind a metabolic response to environmental fluctuations? How did plants evolve to cope with adverse environmental conditions?

**Findings:** We were interested in determining the genetic regulation behind the adjustment in lipid metabolism in Arabidopsis after the sudden onset of abiotic stresses (focusing on heat and darkness). The interesting finding of our work is that KCS4, an enzyme involved in the elongation of fatty acids and wax synthesis, determines the differential accumulation of polyunsaturated triacylglycerols (puTAGs). By sequestering the saturated triacylglycerols into wax, KCS4 leaves free puTAGs to accumulate in lipid bodies and be used as an alternative source of energy.

**Next steps:** This approach could further be used to explore the fine-tuning of lipid metabolism in diverse environments and in other plant species.

## Introduction

Plant metabolism profoundly influences or even coordinates signaling, physiological, and defense responses. When the prevailing environment is adverse, biosynthesis, concentration, transport, and storage of primary and secondary metabolites are affected in a stress-dependent manner. These metabolic responses to abiotic stress involve fine adjustments in carbohydrate, amino acid, amine, and lipid pathways. Proper activation of early metabolic responses helps cells restore chemical and energetic imbalances imposed by stress and is crucial to acclimation and survival (Fraire-Velázquez and Balderas-Hernández 2013).

Like many other complex traits, plant metabolism shows extended variation within natural populations and therefore has been widely investigated using genome-wide association studies (GWAS). Genetic factors involved in variation for key metabolic traits have been successfully identified and characterized (i.e. primary and secondary metabolites and enzyme activities) (Wu et al. 2016, 2018; Fusari et al. 2017). A number of GWAS regarding lipid metabolism have been reported and resulted in the identification of new enzymes and regulators in species like Arabidopsis (*Arabidopsis thaliana*), maize (*Zea mays*), coffee (*Coffea arabica*), rice (*Oryza sativa*), and canola (*Brassica napus*) (Riedelsheimer et al. 2013; Gacek et al. 2017; Sant’Ana et al. 2018; Li et al. 2019; Hong et al. 2022). These studies focused on lipid levels under optimal growth conditions. However, the accumulation of lipids is strongly affected by fluctuations in environmental conditions, such as light, temperature, and nutrient availability (Burgos et al. 2011; Caldana et al. 2011; Szymanski et al. 2014). A classic example involves compensation for decreased membrane fluidity under cold stress (Welti et al. 2002). Moreover, adaptation to a high temperature has been associated with an increase in the relative content of digalactosyldiacylglycerol (DGDG) and the ratio of DGDG to monogalactosyldiacylglycerol (MGDG), as well as a moderate increase in fatty acyl saturation of plant lipids (Han 2016).

In plants, the major source of energy in the dark is starch, which is degraded linearly according to the duration of the previous night (Graf and Smith 2011). Therefore, plants subjected to a sudden extended night (dark stress) experience carbon starvation due to limited starch availability. Under these conditions, polyunsaturated fatty acids (PUFA) released from membrane lipids are used as an alternative substrate for respiration via β-oxidation. These released fatty acids are often sequestered into triacylglycerols (TAGs) to avoid the adverse effects of the detergent-like properties of free fatty acids (FFAs). Therefore, it follows that TAG synthesis and lipid droplet formation sometimes constitute a cellular response to lipotoxicity. This sequestration is especially true for PUFA species because they contain multiple double bonds and are liable to produce reactive oxygen species (ROS) (Kao et al. 2018). It has been shown that the process of FFA sequestration into TAGs protects the cell from ROS generated during PUFA peroxidation (Fan et al. 2017).

Major changes in lipidomic composition under stress are accompanied by a remarkable remodeling of gene expression, both at the level of whole metabolic pathways and also involving activation of specific stress-induced isozymes (Szymanski et al. 2014). This work suggests that only by causing a perturbation of the metabolic homeostasis is GWAS likely to capture genes that evolved to stand at critical branch points in the regulation of the lipidome under naturally fluctuating environments.

Here, we performed GWAS using lipidomic data obtained in 4 different environments: control, combined heat and darkness, and 2 different extended darkness conditions. The locus harboring *KCS4*, encoding 3-KETOACYL-COENZYME A SYNTHASE4 (Joubès et al. 2008; Kim et al. 2021), was strongly associated with a high number of polyunsaturated TAGs (puTAGs) in stress conditions but was not detected in control condition. Thus, *KCS4* association is driven by carbon starvation. Using mutants for alternative *KCS4* alleles and ectopic expression in yeast and plants, we explored the biochemical properties of KCS4 and validated its role in lipid metabolism. We place a model for *KCS4* enzymatic and regulatory function, where KCS4 acts as a branch point in the fate of FAs, directing saturated FA to the very long chain fatty acid pathway (VLCFA) and facilitating the pool of polyunsaturated FA in the accumulation of puTAGs. Two *KCS4* alleles have the differential capacities to process saturated FA. Accessions carrying the strong allele show higher levels of VLCFA-derived cuticular waxes and higher levels of puTAGs. In fully exploiting the genetic data available for Arabidopsis, this work sheds light on the evolutionary events shaping the lipidome and further highlights the importance of lipid remodeling in plant response to changing environments.

## Results

### Plasticity in Arabidopsis lipid metabolism under different environmental conditions

To investigate the genetic regulation of lipid biosynthesis in Arabidopsis, we conducted LC-MS-based lipidomic analysis of 301 and 309 Arabidopsis accessions grown in 2 environmental conditions: control (21/16 °C in 16/8-h light/dark cycle, termed CHD) and stress (heat 32 °C + darkness for 24 h, termed HD), respectively. HD was selected as it has previously been shown to have a strong effect on the lipidome (Caldana et al. 2011). The mapping population was phenotyped for 98 and 109 glycerolipids in CHD and HD, respectively (Supplemental Data Set S1).

Principal component analysis (PCA) of lipid data clearly separated the natural ecotypes into 2 different groups according to the 2 environmental conditions tested (Supplemental Fig. S1A). Furthermore, differences in the lipid status between CHD and HD samples showed that TAGs accumulated to higher levels while the levels of monogalactosyldiacylglycerols (MGDGs) and digalactosyldiacylglycerols (DGDGs) mostly decreased under stress (Supplemental Fig. S1B). In addition, there is substantial positive correlation between lipid levels from a given lipid class, suggesting coordinated regulation at the level of lipid classes (Supplemental Fig. S1C). Notably, TAGs showed the highest variation across accessions for both stress and control conditions, with many species falling in the range of >10-fold difference (Supplemental Fig. S1D).

### Genetic basis underlying Arabidopsis lipid metabolism

Lipid intensities from CHD and HD and the genotypic information from the 250 K SNPchip (Horton et al. 2012) were used to perform 2 independent GWAS for the identification of lipid quantitative trait loci (QTL). GWAS revealed 93 and 544 SNP-trait associations in control and stress conditions, respectively, at a genome-wide significance level of LOD ≥ 5.3 (Supplemental Data Set S2). These SNP-trait associations were further grouped into 51 and 66 QTL for CHD and HD conditions, respectively. In total, only 5 QTL were shared between CHD and HD, revealing the great plasticity of the lipidome in response to different environmental conditions (Supplemental Data Set S3).

**Figure 1.**
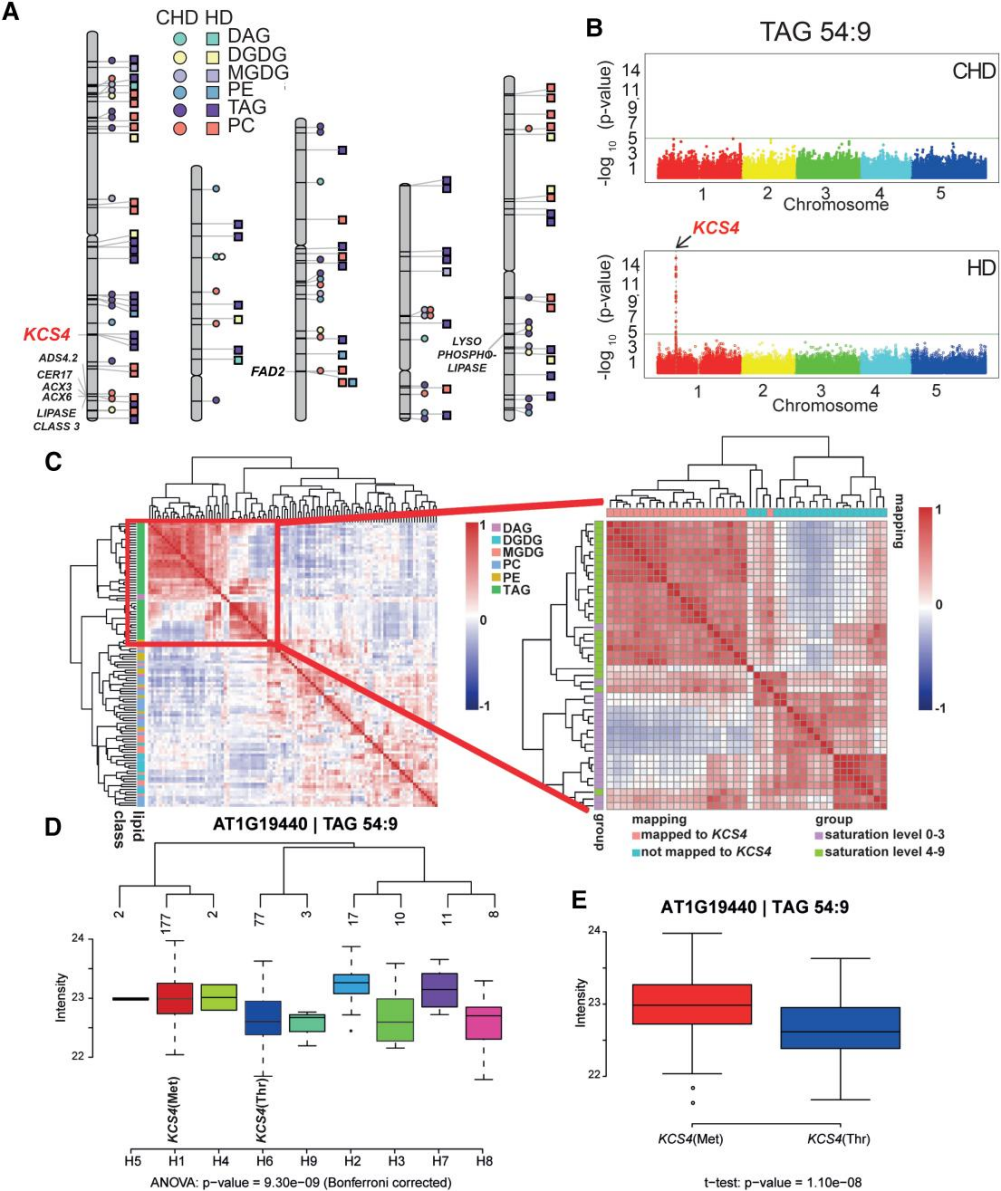
Results from GWAS analysis on lipidomic data from plants grown under control (CHD) and stress (HD) conditions. **A)** Chromosome scheme and QTL identified in the 2 experimental conditions (CHD = circles, HD = squares), for different lipid classes: phosphatidylethanolamine (PE), phosphatidylcholine (lecithin) (PC), MGDG, DGDG, diacylglycerol (DAG), and triacylglycerol (TAG). Color/shape references are included in the figure. Gene names are included only for colocalizing QTL for 2 or more lipid species with LOD > 5.3. **B)** Manhattan plots obtained for TAG 54:9 in CHD (top) and in HD (bottom). The *KCS4* (AT1G19440) QTL was found at Chromosome 1 only under HD condition. **C)** Pearson correlations coefficients calculated between all lipid species across all accessions, for HD condition. Lipids are color-coded according to class (see reference at right) and clustered using Ward’s distance on pairwise dissimilarity. A zoom-in of the TAG cluster shows the grouping pattern of TAGs according to the number of unsaturated bonds and to the GWAS result (left). **D)** Average trait value (intensity of TAG 54:9, log_2_ scale) for the different *KCS4* haplotypes using SNPs m11502, m11503, m11504, and m11505. The 2 major haplotypes have the alternative alleles for m11503 (Met or Thr) **E**. TAG 54:9 is the average value for accessions carrying the *KCS4*(Met) (left) and *KCS4*(Thr) (right) alleles.

Regarding functional annotation of candidate genes, we mapped 12 and 22 genes putatively involved in lipid metabolism for control and stress conditions, respectively (Supplemental Data Set S3, Acyl-Lipid Metabolism database [Li-Beisson et al. 2010] http://aralip.plantbiology.msu.edu/) (Fig. 1). After filtering for robust associations colocalizing for 2 or more traits, 4 QTL harbored genes with annotated functions related to lipid metabolism (Fig. 1A). Two QTL in Chromosome 1, 1 harboring the *KCS4*(AT1G19440) gene and other including a *LIPASE CLASS 3* (AT1G06250), 2 *ACYL-COA OXIDASES* (*ACX3*: AT1G06290 and *ACX6*: AT1G06310) and 2 *FATTY ACID DESATURASE* (*CER17*: AT1G06350, ADS4.2: AT1G063609); 1 in Chromosome 3 harboring the *FATTY ACID DESATURASE* (*FAD2:* AT3G12120) and the last QTL in Chromosome 5, harboring a *LYSOPHOSPHOLIPASE* (AT5G20060) (Supplemental Data Set S3, Fig. 1A).

### *KCS4* is involved in fatty acid elongation (FAE) and has major impact on polyunsaturated TAG levels under heat and dark stress

Remarkably, 22 puTAGs colocalized to the QTL on Chromosome 1, harboring *KCS4* (Fig. 1A). This QTL was not found in control conditions, and the lead SNP in the locus (m11481) colocalized with LOD ≥ 8 (Bonferroni corrected *P*-value < 0.05) for 16 of the 22 associated TAGs (e.g. TAG 54:9, Fig. 1B; Supplemental Data Set S2). Besides *KCS4*, 33 other genes were mapped on the QTL, spanning 107,318 bp (Supplemental Data Set S3). However, with the exception of *KCS4,* none of the others showed any sequence similarity to proteins known to be involved in lipid metabolism.

*KCS4* belongs to a gene family involved in the elongation of VLCFA. Twenty-one *KCS* genes have been previously identified in the Arabidopsis genome. The *KCS* family encodes enzymes involved in the first and often described as rate-limiting step of FAE (Blacklock and Jaworski 2006; Joubès et al. 2008). KCS4's closest homologues are KCS9 (AT2G16280; 78% aa identity), involved in the elongation of C22 to C24 acyl-CoAs (Kim et al. 2013); KCS17 (AT4G34510; 72% aa identity); and KCS18 (AT4G34520; 64% aa identity), involved in elongating C18 acyl-CoAs to C20 and C22 in seeds and shown to drive the natural variation in the abundance of VLCFAs in this organ (Rossak et al. 2001). However, KCS4 specificity and function have not been clearly characterized so far. TAGs associated with the *KCS4* QTL were exclusively polyunsaturated (e.g. puTAG 52:6, 52:7, 54:6, 54:8, 54:9, 56:6, Supplemental Data Sets S2 and S3) and correlated positively with each other, grouping together in a single well-defined cluster (Fig. 1C).

To identify the alleles involved in puTAG variation under HD, we constructed haplotypes considering the 4 SNPs from *KCS4* scored in the 250 K SNPchip (m11502, m11503, m11504, m11505, Supplemental Data Set S2). The population was divided into 9 nonunique haplotypes, which were, therefore, further considered as being putatively involved in the QTL variation. Mean levels of puTAG 54:9 varied significantly among haplotypes (Fig. 1D). Furthermore, m11503 was identified as a nonsynonymous SNP (nsSNP). In the majority of accessions, nsSNP m11503 encodes for a methionine (hereafter *KCS4*[Met]) Accessions carrying this allele have higher levels of puTAG in the stress condition than accessions carrying the minor allele, which encodes for a threonine (hereafter *KCS4*[Thr]) (Fig. 1E). These results suggest that the nsSNP (m11503) in *KCS4* is the causal mutation underlying puTAG variation under HD conditions in Arabidopsis.

### *KCS4* associates with puTAGs in extended darkness

Thus far, we found that *KCS4* associated with puTAGs in a combined heat and darkness treatment. In prolonged darkness, starch is prematurely exhausted and plants are carbon-starved (Stitt and Zeeman 2012). Therefore, we determined whether *KCS4* also associates with puTAGs, when darkness alone is applied.

For this purpose, we utilized our own data sets: control (CD), 3 days (3D) extended darkness, and 6 days (6D) extended darkness (Supplemental Data Set S1) (Zhu et al. 2022). The GWAS at a genome-wide significance level of LOD ≥ 5.3 resulted in 230, 255, and 342 SNP-trait associations grouped into 73, 81, and 70 QTL for CD, 3D, and 6D, respectively (Supplemental Data Sets S2 and S3). The locus harboring *KCS4* was associated strongly in 3D and 6D with 8 and 13 puTAGs, respectively. Lead SNP and other features of the association matched with GWAS results from the HD experiment (Supplemental Fig. S2). Thus, darkness alone is enough to trigger the association of *KCS4* with puTAGs and could be related to the carbon status of the plant. Expression of *DIN1* (AT4G35770) and *BCAT2* (AT1G10070), 2 genes usually used as starvation markers (Moraes et al. 2019) is upregulated under HD conditions (Supplemental Fig. S3), showing that HD is also triggering carbon starvation. In summary, an additive effect is observed when both stresses are applied together, determining a stronger association of *KCS4* to puTAGs under HD.

### Dissection of *KCS4* alleles involved in TAG variation, pattern of linkage disequilibrium (LD), and signatures of selection

**Figure 2.**
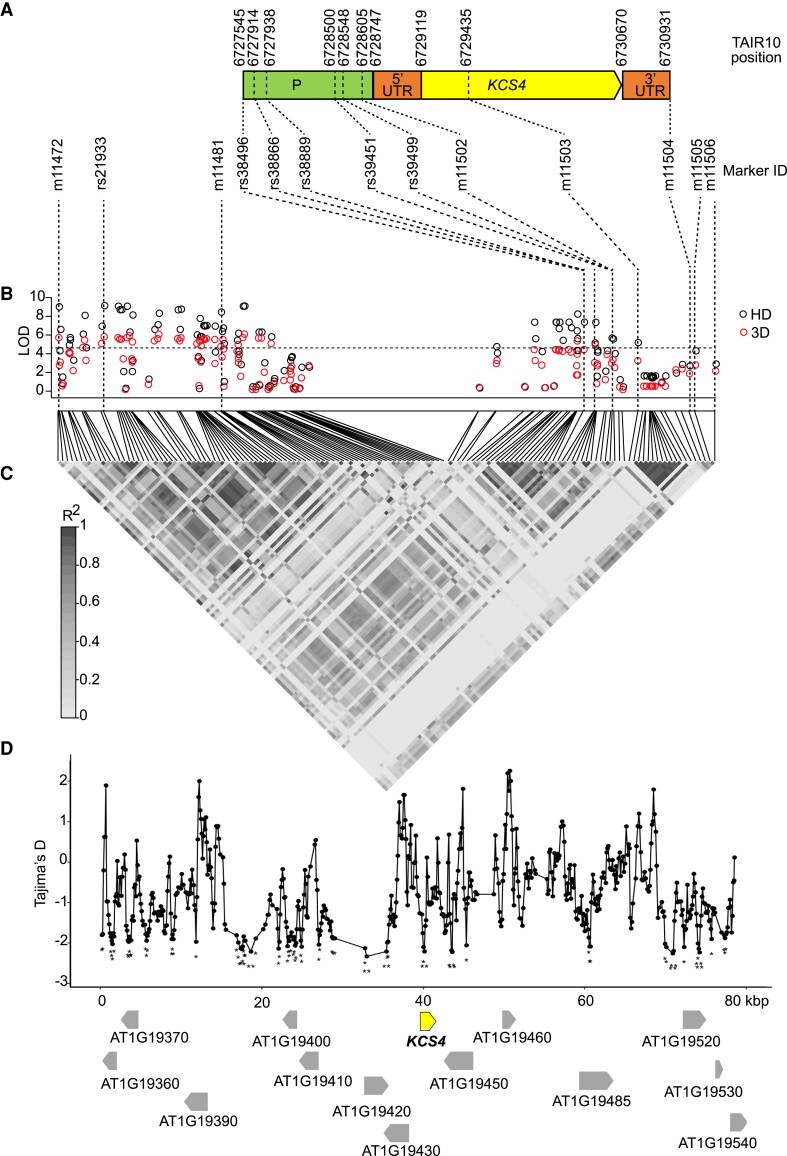
Dissection of *KCS4* alleles involved in TAG variation, pattern of LD, and signatures of selection. **A)***KCS4* gene model depicting promoter region (P), 5′UTR, coding region (*KCS4*), and 3′UTR of *KCS4* (AT1G19440). Beginning, end and relevant SNP positions are indicated above (TAIR10) and below (SNP ID) gene structure. **B)** Manhattan plot of GWAS results (HD: black circles; 3D: red circles) obtained for TAG 54:9 using re-sequenced information for 149 accessions. The re-sequenced region on Chromosome 1 (positions 6689119 to 6769118, TAIR10) included 131 SNPs genotyped in the 250 K SNPchip plus 1,137 additional polymorphisms. Bonferroni threshold (LOD = 4.4) is indicated with a horizontal dashed line. **C)** LD is depicted as a heat map of the coefficient of correlation (*r*^2^). The LD block (0.8 > *r*^2^ > 0.2, *P* < 0.001) includes the lead SNPs rs21933 and m11481, SNPs in *KCS4* promoter (rs39451, rs39499, m11502), in *KCS4* genomic region (m11503) and in *KCS4* 3′UTR (m11504). **D)** Tajima's *D* value in sliding windows for the genomic region analyzed in **(B)**. Gene models and gene orientation are depicted with arrows, *KCS4* (in bold) is highlighted. Significantly negative Tajima's *D* values are marked with asterisks (*P < 0.05, **P < 0.01).

We next mined the full sequence information available for 149 accessions from the GWAS population (http://signal.salk.edu/atg1001/3.0/gebrowser.php), for 80 kb around SNP m11481 on Chromosome 1. The sequence alignment (Supplemental File S1) was used to build an SNP matrix (Supplemental Data Set S4) to perform GWAS with more SNPs than those included in the previous experiments using the 250 K SNPchip (Horton et al. 2012). We analyzed a total of 1,082 SNPs and short insertion–deletions, in addition to the 131 SNPs previously tested in this region. These analyses aim to discard genetic heterogeneity among other genes in the locus, analyze the pattern of LD in the region, determine whether more SNPs in *KCS4* are additionally involved in the variation, and evaluate signatures of selection. The results are shown in Fig. 2, exemplified for TAG 54:9. According to the *KCS4* gene model (Fig. 2A), we identified 35 additional SNPs in the *KCS4* genomic region apart from the 4 SNPs scored in the 250 K SNPchip (19 in the promoter, 2 in the 5′UTR, 13 in the coding region, and 1 in the 3′UTR; Supplemental Data Set S5). Fewer TAGs with lower LOD values showed an association with *KCS4*, as expected when analyzing fewer accessions (<100) (Supplemental Data Set S5, Fig. 2B). The lead SNP using resequencing information was a polymorphism not tested before (rs21933), but in high LD with m11481 (*r*^2^ = 0.73, *P* < 0.001). In addition, a number of polymorphisms in the upstream region of *KCS4,* not tested in the SNPchip, were identified as being significant, pointing to a putative combined regulation at the promoter region mediated by rs39451, rs39499, and m11502 and at the coding region mediated by the nsSNP m11503 (Fig. 2A). Surprisingly, several SNPs in the *KCS4* coding region did not associate (Fig. 2B).

All SNPs in the locus harboring *KCS4* with high LOD values are in high LD with each other and in high LD with the lead SNPs rs21933 and m11481 (*r*^2^ > 0.2, *P* < 0.001, Fig. 2C). The remaining SNPs in the *KCS4* coding region are not in LD with the lead SNPs nor with the nsSNP m11503 in the *KCS4* coding region. This result suggests that these mutations may have appeared at a different time during evolution or they may have accumulated later, as a consequence of specific recombination, to increase allele frequency at other SNP positions. To evaluate signatures of selection, we tested Tajima’s *D* for the 80 kb around m11481 (Fig. 2D). Tajima’s *D* reached significantly negative values along the 80 kb, particularly around SNPs significantly associated with the QTL: rs21933, m11481, rs39451, rs39499, m11502, m11503. This result indicates that the region is under high selective pressure and supports the selection of *KCS4* as the candidate gene involved in puTAG variation.

### Transcriptional regulation of *KCS4* in Arabidopsis

**Figure 3.**
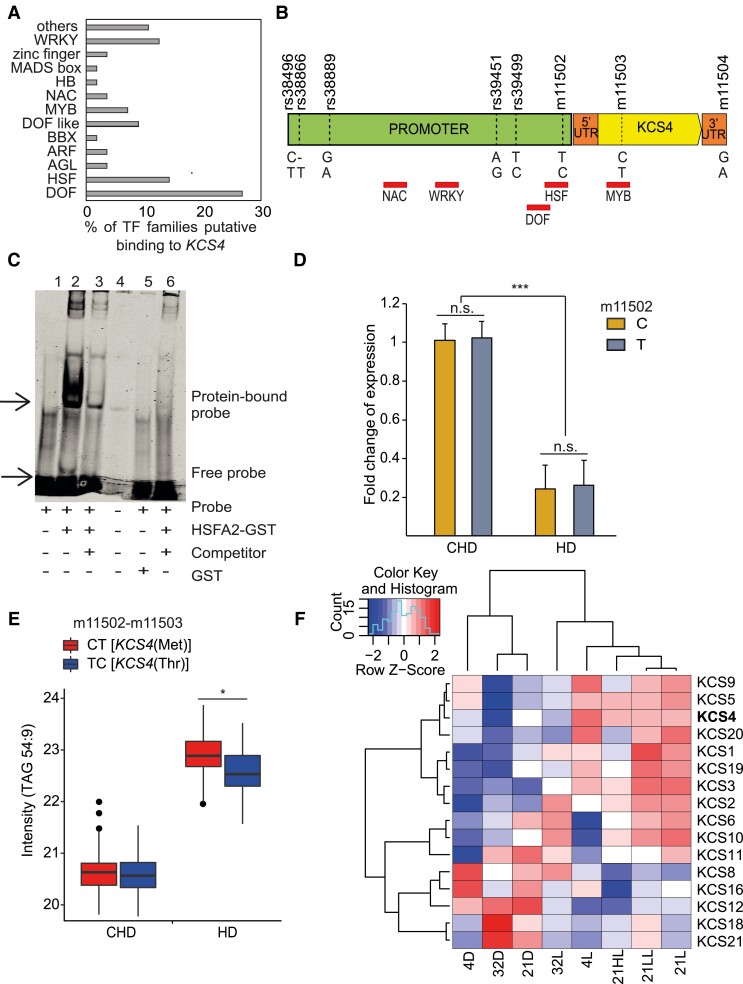
KCS4 transcriptional regulation. **A)** Percentage of TFs classes predicted to bind *KCS4* genomic region according to in silico analysis of binding motives using DAP-seq database (O’Malley et al. 2016). **B)** Position of the binding motifs (red rectangles, names below) for the main TF classes. SNP positions in the promoter and in coding region are marked with vertical dash-lines and IDs are stated above gene model. **C)** EMSA showing the interaction between *HEAT SCHOCK FACTOR A2* (*HSFA2*/AT2G26150) and the HSE binding motif (heat shock element: 5′-nnGnAnnTnCtn-3′) found within the SNP m11502 in *KCS4* promoter region; 1: labeled probe (5′-DY682-labeled double-stranded oligonucleotide) only; 2: labeled probe plus HSFA2-GST protein; 3: labeled probe, HSFA2-GST protein and 200× competitor DNA (unlabeled oligonucleotide containing HSE binding site with the “C” allele at m11502), 4: well spill, 5: labeled probe plus free GST, 6: labeled probe, HSFA2-GST protein and 200× competitor DNA (unlabeled oligonucleotide containing HSE binding site with the “T” allele at m11502). **D)***KCS4* expression for the complete GWAS population in CHD and HD conditions, grouped based on C or T allele at SNP m11502 in *KCS4* promoter region. Expression (fold change) was measured by RT-qPCR. Significant differences between CHD and HD transcript levels and between C and T alleles are shown (*t*-test: *P < 0.05, **P < 0.001, ***P < 0.0001, n.s. not significant). Data represent means ± Sd. For CHD *n* = 193 for the C allele and *n* = 94 for the T allele. For HD *n* = 163 for the C allele *n* = 75 for the T allele. **E)** Average trait value (TAG 54:9 intensity, in log_2_) for the 2 major haplotypes determined by SNPs m11502 (promoter) and m11503 (coding region). CT and TC haplotypes encodes for *KCS4*(Met) and *KCS4*(Thr), respectively. Significant differences between haplotypes (*t*-test: *P < 0.05). **F)** Heatmap of expression pattern for 16 members of the *KCS* multigene family clustered using complete-average method. Transcript abundance was measured 24 h after treatment. Data used in **F** was obtained previously (Caldana et al. 2011).

Several SNPs in the promoter region showed significant LD with the nsSNP m11503. Therefore, we focused on the transcriptional regulation of *KCS4* in Arabidopsis (Fig. 3). We analyzed 1 kb upstream of the transcription start site for known transcription factor (TF) binding sites using the DAP-Seq database (O’Malley et al. 2016). Several TF families belonging to land plants could likely bind to the *KCS4* promoter region, indicating that *KCS4* expression is controlled by several regulatory processes (Fig. 3A). We furthered our analysis by looking at the core binding sites of the highly enriched TF families that contained binding motifs across SNPs in the promoter region of *KCS4* (rs38889, rs39451, rs39499, and m11502). The top-enriched DOF family (DNA-binding One zinc Finger) does not have a binding site colocalizing with polymorphisms but does in other regions of the promoter (Fig. 3B). Similarly, the NAC and WRKY TF families bind to nonpolymorphic regions in the *KCS4* promoter, and MYB TF binds near an SNP but in the coding region. Interestingly, heat shock factor (HSF), the second highly enriched TF family, has a core binding motif (heat shock element: 5′nnGnAnnTnCtn 3′) within SNP m11502 in the promoter region.

We performed an electrophoretic mobility shift assay (EMSA) to assess the physical interaction of HSFA2 (AT2G26150) with the *KCS4* promoter sequence. Additionally, we tested if the alleles (C and T) at SNP m11502 influence the binding. HSFA2, a well-studied HSF family member, has been shown to bind to the heat shock element in Arabidopsis (Lämke et al. 2016). As shown in Fig. 3C, HSFA2-GST fusion protein bound to the 5′-DY682-labeled *KCS4* promoter fragment in vitro with the C allele at m11502, resulting in a retardation band on the gel (Fig. 3C, Lane 2) compared to the free probe (Lane 1). Interaction between HSFA2 and the *KCS4* promoter region containing the binding site appears to be specific, as the addition of an excessive unlabeled competitor (same sequence, 200× more) significantly reduced the binding to the labeled probe (Fig. 3C, Lane 3). Finally, we tested a competitor sequence having the alternative allele at m11502 (T). The latter reduced the binding compared to the labeled probe (Fig. 3C, Lane 6), suggesting that the T allele at m11502 promotes stronger binding of HSF2A to the *KCS4* promoter region. Overall, this indicates that polymorphisms at the promoter region could exert a dosage effect on TFs, influencing the expression of *KCS4*.

Furthermore, we analyzed natural variation in transcript levels of *KCS4* for the CHD and HD conditions in the complete GWAS panel (Fig. 3D). *KCS4* transcripts were significantly lower in HD compared to CHD. This correlates significantly negatively with highly unsaturated TAG levels (*r*^2^ = −0.66, *P* < 0.001), which increased in HD (e.g. Fig. 3E, TAG 54:9). In HD, *KCS4* transcript levels were not significantly different for accessions carrying different alleles at m11502. This confirms that neither differences in TF binding affinity nor expression levels of *KCS4* determine the variation of puTAG levels under HD (Fig. 3E). In other words, polymorphisms at the *KCS4* promoter region are not causal for puTAG variation in Arabidopsis natural populations.

### Expression patterns of *KCS* family members under diverse environmental conditions

We analyzed the time-resolved response of Arabidopsis Col-0 accession towards light and temperature for 16 of the 21 gene members using data previously obtained (Caldana et al. 2011). Focusing on 24 h, most *KCS* members, including *KCS4*, are upregulated under light conditions and downregulated in darkness, irrespective of the temperature. However, *KCS8* (AT2G15090), *KCS12* (AT2G28630), *KCS16* (AT4G34250), *KCS18*, and *KCS21* (AT5G49070) showed the opposite pattern. *KCS4* transcript levels were downregulated both under darkness and under HD, but transcript levels were much lower in HD, strengthening the idea of a synergistic effect between light and temperature stress in the *KCS4* response. In addition, *KCS5* (AT1G25450), *KCS9*, and *KCS20* (AT5G43760) showed a similar transcriptional response to *KCS4* across several environmental stimuli (Fig. 3F).

### Functional validation of the role of *KCS4* in puTAG variation

**Figure 4.**
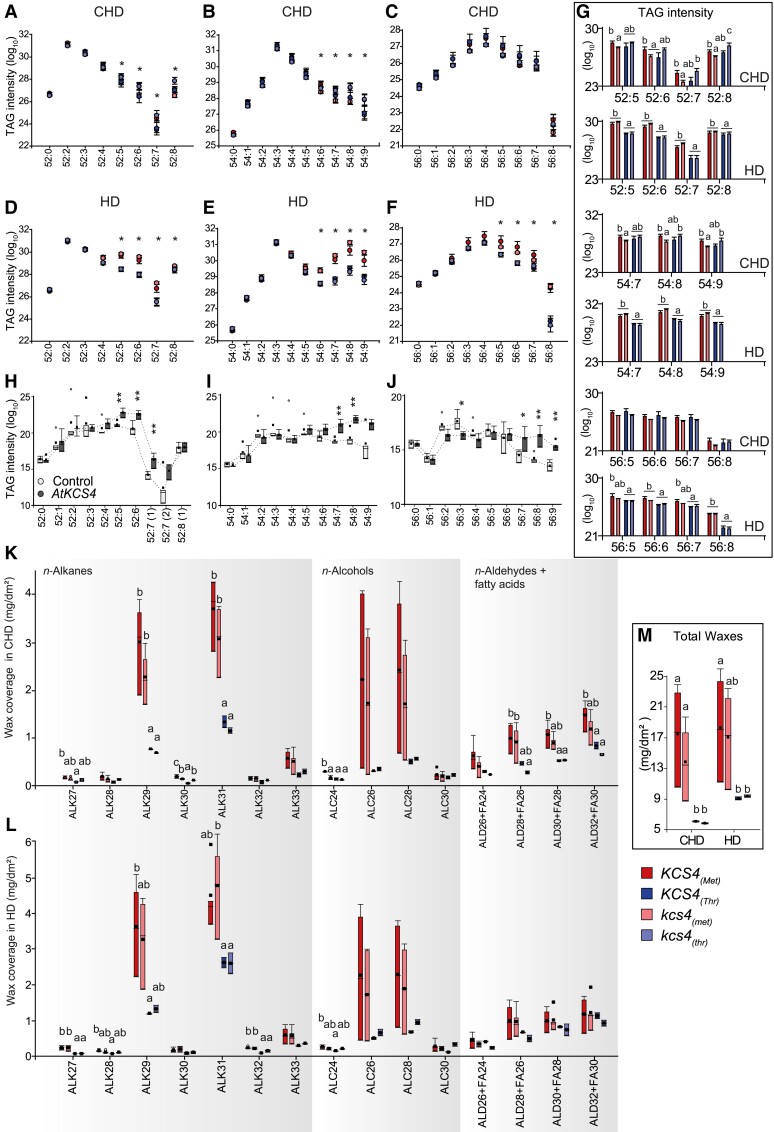
*KCS4* functional validation using allelic mutants in Arabidopsis and heterologous expression in *N. benthamiana.***A–G)** Average TAG profiles for wild-type (WT) accessions and allelic mutants carrying the *KCS4*(Met) allele (WT: red; mutant: pink) and the *KCS4*(Thr) allele (WT: blue; mutant: light-blue) in CHD and HD conditions. Dot plots **(A–F)** and bars **(G)** represent means ± Se, *n* = 14 to 25. One-way ANOVA followed by Fisher-Lsd post hoc test was performed to detect differences between lines for each TAG species. Significant changes (*P* < 0.05) are marked with asterisks **(A–F)** or different letters **(G)**. **H–J)** Transient expression of At*KCS4*(Met) in *N. benthamiana* leaves. Plants were grown in CHD conditions for the whole experiment. TAG levels for classes 52 (52:0 to 52:8), 54 (54:0 to 54:9) and 56 (56:0 to 56:0) for the control line (white) and for the At*KCS4*(Met) line (dark grey). Box-plots (*n* = 3). Significant values (*t*-test) are indicated (*P < 0.05, **P < 0.01). **K–M)** Leaf cuticular wax coverage for WT accessions and allelic mutants carrying the *KCS4*(Met) allele (WT: red; mutant: pink) and the *KCS4*(Thr) allele (WT: blue; mutant: light-blue) in CHD and HD conditions. ALK: alkane, ALC: alcohol, ALD: aldehyde, FA: fatty acid. Box-plots (*n* = 4). One-way ANOVA followed by Fisher-Lsd post hoc test was performed to detect differences between lines for each wax species. Means with a common letter are not significantly different (*P* < 0.05).

To validate the role of *KCS4* alleles in puTAG variation under HD stress, we selected 5 accessions: 3 carrying *KCS4*(Met) and 2 carrying *KCS4*(Thr). Selection of these accessions was based on puTAG levels measured in the HD experiment (Supplemental Data Set S1). Mutant lines (*kcs4*) were obtained through CRISPR-Cas9 by introducing a 57/58-bp deletion at position +18 from the ATG (Supplemental File S2). This deletion introduced a premature stop codon, resulting in a truncated protein of 16 aa compared to the wild-type version of 516 aa. Lines were grown under CHD or HD as described in materials and methods and harvested to perform lipidomic analysis. Since natural variation is detected at a population level, we compared the average values for *kcs4*(Met) and *kcs4*(Thr) allelic mutants against wild-type *KCS4*(Met) and *KCS4*(Thr) alleles (Fig. 4).

Under control conditions (CHD), puTAG levels (X:4 to X:9) were significantly different between *KCS4*(Met) and *kcs4*(Met) lines. Mutants for *kcs4*(Met) presented, on average, lower levels of puTAGs, similar to the levels found for *KCS4*(Thr) accessions (Fig. 4, A–C and G). This phenomenon was seen for the accessions pooled together as well as for individual accessions (Supplemental Data Set S6). On the other hand, puTAG levels did not differ between *KCS4*(Thr) and *kcs4*(Thr). These results support *KCS4*(Met) as the stronger allele and the causal polymorphism for polyunsaturated TAG variation.

Under HD condition, a higher accumulation of puTAGs was seen for accessions carrying *KCS4*(Met) allele (Fig. 4, D–G). However, overall levels of TAGs (saturated and polyunsaturated) increased independently of *KCS4* alleles. This is in agreement with the phenotype observed for the complete mapping panel. Here, mutants didn’t differ in puTAG levels from their wild-type counterparts, as if the massive production of puTAGs under stress overrides the effect of *KCS4* mutation.

Transient expression of At*KCS4*(Met) in *Nicotiana benthamiana* leaves showed significantly higher levels of puTAGs for classes 52, 54, and 56 compared to control plants (Fig. 4, H–J). Conversely, TAGs with none or a few unsaturated bonds tended to be higher in control plants. These results show that the transient expression of At*KCS4*(Met) changes the TAG profile in *N. benthamiana*.

In summary, the reduction of puTAGs in the *kcs4*(Met) mutant (Fig. 4, A–C and G), together with the increase of puTAGs when *KCS4*(Met) was expressed in *N. benthamiana* (Fig. 4, H–J), confirms that *KCS4*(Met) is the causal polymorphism for polyunsaturated TAG variation and validates GWAS results.

### Functional validation of the role of *KCS4* in the accumulation of leaf cuticular waxes

VLCFAs are produced by the elongation complex (i.e. KCS, KCR: β-KETOACYL REDUCTASE, HCD: β-HYDROXYACYL-COA DEHYDRATASE and ECR: ENOYL-COA REDUCTASE) in the endoplasmic reticulum (ER) and used as precursors for the synthesis of lipid barriers such as cuticular waxes. Therefore, we determined the leaf wax coverage for *KCS4* wild-type alleles and mutants as a proxy of the enzyme activity in planta (Fig. 4, K–M). In general, lines carrying *KCS4*(Met) showed significantly higher content of major components of leaf waxes than lines carrying *KCS4*(Thr) in both CHD and HD conditions (Fig. 4M). Particularly for CHD condition, the mutant *kcs4*(Met) showed a significant reduction of the compounds ALK30 and ALC24 compared to the wild-type allele *KCS4*(Met). Other wax compounds such as ALK27, ALD30 + FA28, and ALD32 + FA30 showed the same tendency, although values were not significantly different from the wild-type *KCS4*(Met) allele (Fig. 4K). On the other hand, *kcs4*(Thr) did not show any differences from its wild-type counterpart. These results confirm that the wax coverage and composition are different for both alleles and that other members of the KCS family cannot fully complement the synthesis of waxes coming from FA elongated by KCS4.

### KCS4 enzymatic activity and cellular localization

*KCS4* shows sequence similarity to the remaining 20 members of the Arabidopsis *KCS* multigene family (Joubès et al. 2008). As stated before, KCS enzymes catalyze the first rate-limiting step in the VLCFA elongation, and they determine the substrate specificity.

**Figure 5.**
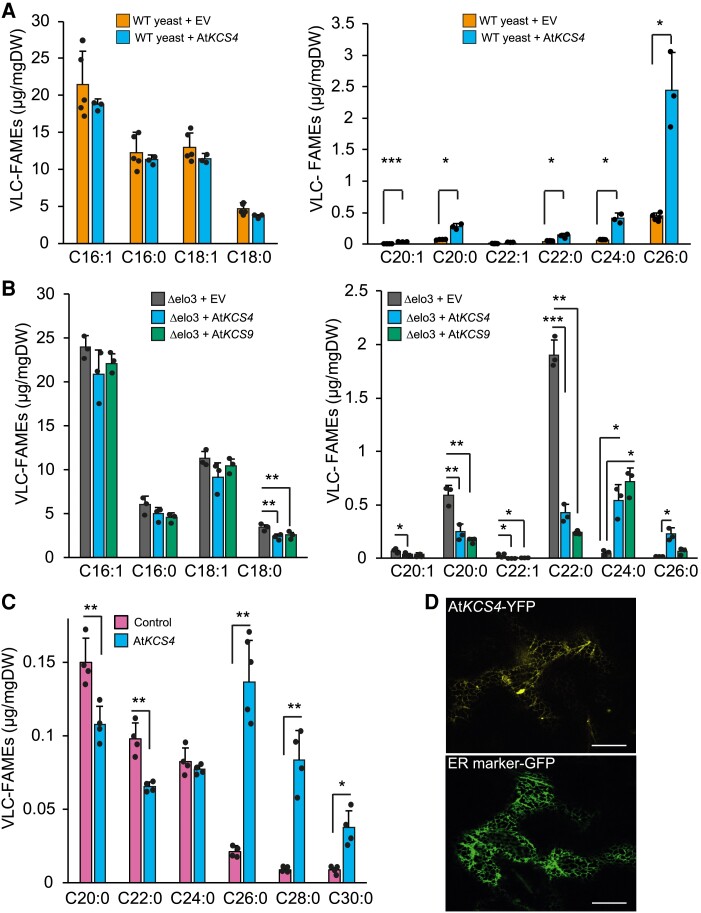
KCS4 activity and specificity in FAE and subcellular localization. **A)** Very long chain fatty acid methyl ester (VLC-FAME) profiles (16 to 18 and 20 to 26, separate panels) of WT yeast transformed with an empty vector and *AtKCS4*(Met) (reference included in the figure). **B)** VLC-FAME profiles (16 to 18 and 20 to 26, separate panels) of Δelo3 yeast mutant transformed with an empty vector (EV, grey), *AtKCS4*(Met) (blue), and *AtKCS9* (green). **C)** VLC-FAME profiles (20 to 30) from leaves of *N. benthamiana* control plants or transformed with *AtKCS4*(Met). Bars represent means ±Sd (*n* = 3 to 5), individual values are plotted as filled circles. Significant differences for *t*-test are shown (*P < 0.05, **P < 0.01, ***P < 0.001). **D)** Confocal images of *N. benthamiana* leaves transiently transformed with KCS4(Met)-YFP construct. The plasmid ER-GK CD3-955 was used as an ER marker. Scale = 20 *µ*m. DW: dry weight.

To evaluate the very-long-chain 3-ketoacyl-CoA synthase activity and substrate specificity of KCS4, we expressed *AtKCS4*(Met) in wild-type yeast and profiled VLCFAs (Fig. 5). The strain expressing *AtKCS4*(Met) synthesized significantly higher levels of VLCFA from C20 to C26 compared to the strain transformed with an empty vector (Fig. 5A).

We further expressed *AtKCS4*(Met) in the Δ*elo3* yeast strain, lacking the endogenous ELO3 elongase (Fig. 5B). The Δ*elo3* strain accumulates C20 and C22 VLCFA (Han et al. 2002). Expression of KCS4 led to the elongation of VLCFAs from C22 to C24 and from C24 to C26 (Fig. 5B). We also analyzed the heterologous expression of At*KCS9* as it is the closest *KCS4* homolog (Fig. 5B). KCS9 elongates VLCFAs from C22 to C24 (Kim et al. 2013 and Fig. 5B). These results suggest that KCS4 has differential activity compared to KCS9, pushing further elongation of VLCFAs up to C26.

Transient expression of *AtKCS4*(Met) in *N. benthamiana* leaves produced a significant decrease of C20 and C22 VLCFAs and a significant increase of VLCFAs from C26 up to C30 compared to the control (Fig. 5C). The elongation of VLCFAs up to C30 might be the result of the action of the native elongase complex in *N. benthamiana* due to the increased availability of C26 when *KCS4* is transiently expressed. AtKCS4(Met)-YFP was found to label a polygonal and tubular network characteristic of the ER (Fig. 5D), in agreement with previous results shown for other KCS family members (Joubès et al. 2008).

We further transformed Δ*elo3* strain with *KCS4* wild-type and mutant alleles (Supplemental Fig. S4). Expression of both *KCS4*(Met) and *KCS4*(Thr) alleles led to the elongation of C22 into C24 up to C26 VLCFA in the Δelo3 strain with slight differences in the amount of C22 and 2OH:C24 (Supplemental Fig. S4A). On the other hand, yeast expressing allelic mutants did not show exactly the same composition as the control line, C26 did not accumulate, but C22 and C24 VLCFA were still produced at a low level compared to yeast expressing wild-type alleles as if some activity remained. This residual accumulation of VLCFA in the mutants indicates that *kcs4*(Met) and *kcs4*(Thr) lines still have some enzymatic activity, although very weak.

In addition, we performed a time-course experiment at 48, 72, and 96 h for Δ*elo3* strain expressing *KCS4*(Met) *and KCS4*(Thr) wild-type alleles (Supplemental Fig. S4B). Yeasts expressing *KCS4*(Met) ended up with more C26 and 2OH:C26 than yeasts expressing *KCS4*(Thr), supporting the hypothesis of a differential enzymatic activity between *KCS4* alleles.

KCS1 (AT1G01120) expression in yeast leads to the production of C18, C20 and C22 VLCFA (Trenkamp et al. 2004; Tresch et al. 2012), KCS2 (AT1G04220) can elongate from C20 to C22 (Millar et al. 1999; Franke et al. 2009), KCS20 mainly produces C22 and C24 VLCFA, and KCS5 and KCS6 (AT1G68530) mainly produce C24 to C28. All these proteins have different expression profiles under heat and dark stress, with some being upregulated and some being downregulated (Fig. 3F). Expression profiles of *KCS4*, *KCS5*, *KCS9*, and *KCS12* are also altered in *kcs4* CRISPR-Cas lines, evidencing a delicate expression balance under stress, between KCS family proteins (Supplemental Fig. S5).

### Biomass is affected by KCS4 function

**Figure 6.**
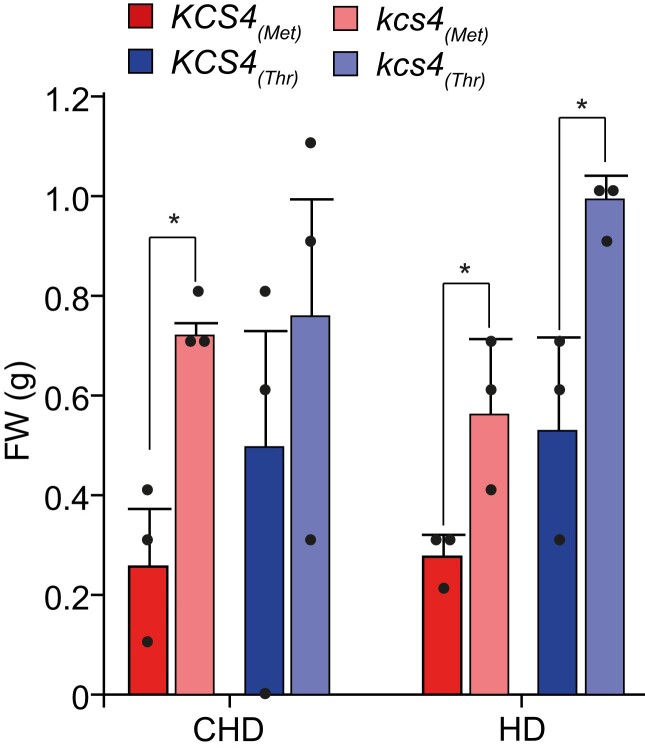
Biomass changes in *KCS4* WT and allelic mutants. Rosettes fresh-weight measured in accessions carrying *KCS4*(Met) allele (WT: red, mutant: pink) or *KCS4*(Thr) allele (WT: blue, mutant: light-blue). Plants were grown in control conditions (CHD) or subjected to heat and darkness for 24 h (HD). Data represent means ± Se, *n* = 3. Significant differences for *t*-test are shown (*P < 0.05).

A mild reduction in VLCFA content improves cell proliferation and shoot growth (Nobusawa et al. 2013). To investigate if *KCS4* has an effect on plant growth, we compared biomass of *KCS4* wild-type accessions and mutant lines. We collected full rosettes from plants grown in CHD and subjected to HD, immediately after treatment. The *kcs4* mutants grew significantly bigger than their WT counterparts (Fig. 6). This indicates that biomass decreases with increasing KCS4 activity and further supports both the idea that mutations at *KCS4* reduce the overall content of VLCFA and that KCS4 activity cannot be overcome by other family members.

### Phylogenetic signal of the lipidome and correlation with climatic and geographical conditions

To test if there is a correlation between lipid profiles and genetic distance (a proxy for phylogeny), *K* statistics and empirical *P*-values were calculated for all the measured lipid features in control (CHD) and stress (HD) conditions (Blomberg et al. 2003). The results indicate a correlation between genetic distance and the similarity of lipidomic profiles. Lipids exhibiting a significant phylogenetic signal in CHD are enriched in MGDGs (*P* ≤ 0.001), while in HD, we observed enrichment in TAGs (*P* ≤ 0.05). Five of the 6 TAGs with a significant phylogenetic signal are polyunsaturated species associated with the *KCS4* QTL (Supplemental Fig. S6).

In addition, climate conditions from the geographical origins of accessions (Fick and Hijmans 2017) were also analyzed to identify putative correlations with lipidomic profiles. We used a lasso-regularized linear model to predict each climate parameter from the lipidomic profiles. In result, we estimated the prediction power for each best-fitting model, and the lipids, being the best predictors for the climate parameters. While the CHD condition did not provide significant results, the HD condition highlighted in total 15 significant model fits, 5 of which also gave significant results in a cross-validation test (Supplemental Fig. S7). All of these 5 significant variables are related to temperature. In total,6 “climate-predictive lipids” were also significantly associated with *KCS4*, with TAG 58:6, TAG 60:6, and TAG 56:8 being linked to the temperature in the driest and the coldest quarters of the year.

**Figure 7.**
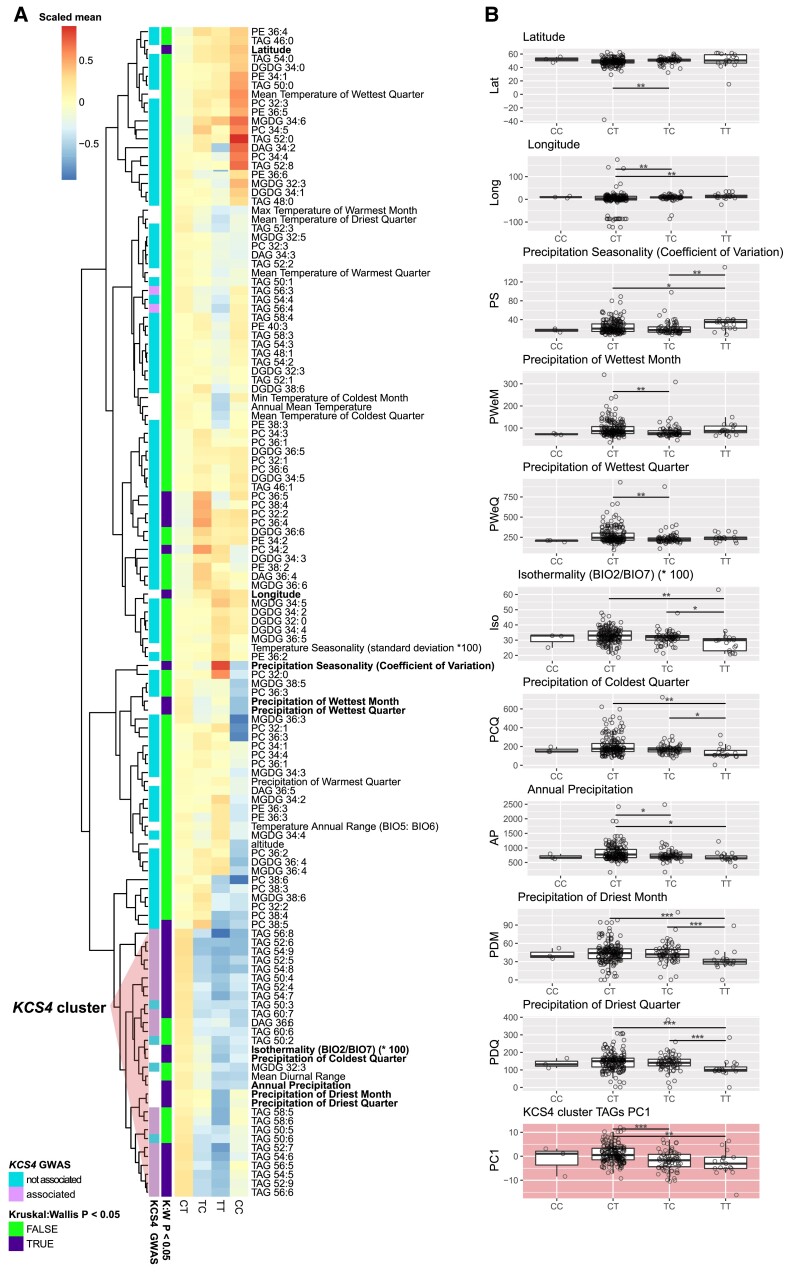
*KCS4* haplotype correlation with climate and geographic parameters. **A)** Heatmap of the mean values of lipid, climate, and geographical variables for each of the 4 *KCS4* haplotypes (SNPs m11502-m11503). Results of the *KCS4* locus association as well as nonparametric ANOVA test (Kruskal–Wallis) are highlighted in the row annotation bar. Cluster dominated by KCS4-associated TAGs is marked. **B)** Average haplotype value for the principal component 1 (PC1) obtained using the data in KCS4 cluster (in **A**), and the climate and geographical parameters showing significant Kruskal–Wallis in **A**. Significant differences are shown (pairwise Wilcoxon rank sum test: *P < 0.05, **P < 0.01, ***P < 0.001).

Finally, cluster analysis was performed for climate parameters and TAG levels by grouping accessions according to *KCS4* haplotypes (m11502-m11503: CC, CT, TT, TC), and significant differences between mean values for haplotypes were tested using nonparametric ANOVA (Fig. 7). Eight climate parameters plus latitude and longitude differed significantly between haplotypes (*P* < 0.05, Fig. 7A). Moreover, 5 of them coclustered with TAGs associated with *KCS4*, 4 of them related to precipitation, and 1 to temperature (isothermality). The “TT” haplotype in most cases, presented the lowest value for these parameters, suggesting that the allelic effect of these 2 SNPs is opposite for climate variables (Fig. 7B).

## Discussion

Recent years have witnessed the successful application of metabolic GWAS (Luo 2015). In lipids, the effort has focused more on dissecting the genetic architecture of oil biosynthesis (Correa et al. 2020). Here, we focus on glycerolipid profiles of ∼300 Arabidopsis natural accessions grown under control conditions and subjected to a sudden environmental change given by combined stress of heat and darkness. Our GWAS exposed the high plasticity of the Arabidopsis genome, allowing the identification of genes involved in the regulation of lipid metabolism, in an environment-dependent manner (Fig. 1A).

Lipid-based GWAS in control conditions showed several mild-strength associations, similar to what was found for glycerolipids in rice (Hong et al. 2022) and for primary metabolites in maize and Arabidopsis (Wen et al. 2015; Wu et al. 2016), which are controlled by a large number of small-effect loci. This could be explained by the fact that both lipids and primary metabolites are essential for the survival of the cell and thus, do not show on/off phenotypes. This is different from natural variation in secondary metabolites, which is controlled, to a large extent, by major large-effect loci (Chan et al. 2011; Matsuda et al. 2012; Wen et al. 2014; Wu et al. 2018).

In our study, applying combined heat and darkness resulted in a strong perturbation of lipid homeostasis. This led to the identification of a large-effect QTL at Chromosome 1, harboring *KCS4*. *KCS4* is associated with several puTAGs specifically under carbon starvation conditions (HD, 3D, and 6D) in a highly robust manner (Fig. 1B, Supplemental Fig. S2B and Data Sets S2/S3). Under HD, all puTAGs mapping to *KCS4* are higher than under CHD, but accessions carrying *KCS4*(Met) always have higher levels of puTAGs than accessions carrying *KCS4*(Thr). PuTAGs that mapped to *KCS4* clustered together and correlated negatively to saturated TAGs, further illustrating the role of *KCS4* in the specific regulation of puTAGs (Fig. 1C).

We have provided several lines of evidence that *KCS4* is the causal gene for TAG variation and that the *KCS4*(Met) allele determines higher levels of puTAGs under stresses (HD, 3D, 6D): (i) LD, correlation among lipid species and haplotypic analysis point *KCS4* as the candidate gene for puTAG variation under stress (Figs. 1 and 2), (ii) transient overexpression of *KCS4*(Met) in *N. benthamiana* mimics the phenotype we observed in the GWAS population grown under stress conditions (Fig. 4, H–J), (iii) lipidomic analysis of allelic mutants (*kcs4*[Met]/*kcs4*[Thr]) and their respective wild-type accessions (*KCS4*[Met]/*KCS4*[Thr]) shows a clear phenotype on puTAG, VLCFA, and leaf wax components (Figs. 4 and 5, Supplemental Fig. S5). Moreover, our detailed expression analysis of the entire population indicates that TAG variation in HD conditions is not due to differences in the expression of *KCS4* alleles (Fig. 3D). This finding further supports the hypothesis that the causal polymorphism is the nsSNP m11503, leading to a Met to Thr amino acid change.

KCS4 is a functional enzyme, localized in the ER, with substrate specificity for VLCFAs of C22 and C24 length, and has the ability to elongate VLCFAs to C24 and up to C26 (Fig. 5). This result is in agreement with Kim et al. (2021) findings for *kcs4* T-DNA mutant, which accumulates C22 and C24. Moreover, the results from ectopic expression in yeast and plants confirmed that KCS4 has specificity for the elongation of saturated FAs (Fig. 5), in agreement with previous findings that showed that elongase-condensing enzymes are mostly active with saturated FA (Blacklock and Jaworski 2006).

Under HD, differences in TAG levels for accessions carrying *KCS4* opposite alleles (*KCS4*[Met] vs. *KCS4*[Thr]) become more significant as the number of double bonds in TAGs increases (Fig. 4, D–G). In addition, TAG levels are significantly different when comparing *KCS4*(Met) with its allelic mutant, *kcs4*(Met), in control conditions (CHD) (Fig. 4, A–C and G). In the same line of evidence, the mutant *kcs4*(Met) showed a significant reduction of the major components of leaf waxes compared to its wild-type allele in CHD (Fig. 4, K–M). Not unexpectedly, *kcs4*(Thr) did not show any differences in TAG levels or wax compounds compared to *KCS4*(Thr). It has been previously shown in GWAS that mutating the weaker allele did not always result in clear phenotypic changes for the traits under study (Todesco et al. 2010; Fusari et al. 2017; Wang et al. 2022; Yang et al. 2022).

The phenotype can also depend on the context, and a change in either the genetic or the environmental background can amplify or remove the effect (Tonsor et al. 2005). Under HD, there is a strong metabolomic and expression reprogramming as shown before for lipids and primary and secondary metabolites (Burgos et al. 2011; Szymanski et al. 2014; Wu et al. 2016, 2018). This could explain why we did not observe a phenotype by mutating only 1 gene. In addition, sometimes effects are not evident when analyzing single mutants, particularly if there are gene families encoding the same or very similar enzymatic activities. For example, in studies of auxin conjugates, indole-3-acetic acid showed a reduction only when triple amidohydrolase mutants were analyzed (Ludwig-Müller 2011). *KCS4* is 1 of 21 genes encoding KCS activity (Joubès et al. 2008; Guo et al. 2016) and we have shown that expression of other *KCS* members is altered in *kcs4* allelic mutants, indicating a complex regulation between *KCS* genes (Supplemental Fig. S5). Of note, the nature of this complex regulation was recently demonstrated to involve protein–protein interactions within the elongase complex (Kim et al. 2022).

During plant evolution, the *KCS* family began with a single gene in algae and evolved via numerous gene duplications into a 21-member gene family in Arabidopsis (Guo et al. 2016). Analysis of LD and Tajima's *D* parameters in the region harboring *KCS4* showed that diversity is limited to 2 major haplotypes and that the region is under strong selective pressure (Fig. 2). As *KCS4* is involved in TAG regulation under conditions where carbon reserves are exhausted, it might be possible that *KCS4* has been targeted for natural selection to enhance adaptation in stressful environments. In nature, 24 h of darkness do not occur, but carbon starvation is also observed when plants are exposed to very limited light periods in regions with high amplitudes of day length. In agreement with the adaptative role of *KCS4* to environmental stresses, the geographical origin of the accessions (i.e. latitude) correlated with *KCS4* haplotypes (Fig. 7). Together, these analyses indicate that polymorphism variation at *KCS4* is important for adaptation in different climatic zones by adjustment of lipid metabolism.

In an event of sudden extended darkness (3D, 6D) or combined heat and darkness (HD), plants become carbon-starved (Supplemental Fig. S3) (Stitt and Zeeman 2012). Increasing night temperatures accelerates enzyme activities, exhausting carbon resources prematurely (Pilkington et al. 2015). In this case, FAs are used as alternative substrates for respiration via β-oxidation (Fan et al. 2014). There are many lines of evidence that support the idea that plant lipids are alternative sources of carbon and energy under stress, particularly under carbon starvation. For instance, Kunz et al. (2009) showed that by knocking out the *PEROXISOMAL ABC-TRANSPORTER1* (*PXA1*, AT4G39850), the import of FFA into the peroxisome is blocked, resulting in plants dying faster in extended darkness. Fan et al. (2017), through the study of *sdp1* mutants (*SUGAR DEPENDENT1*, *SDP1*, AT5G04040), could define the crucial role that TAG metabolism has in membrane lipid breakdown, FA turnover, and plant survival under extended darkness. Another study showed that there are different metabolic strategies employed by leaves in response to different darkening treatments (Law et al. 2018). These strategies involve either importing sugars from sink sources, metabolizing amino acids, or activating the turnover of lipids from the plastid membranes to the β-oxidation in the peroxisome. The last strategy is particularly important after 6 d of dark treatment. Finally, Yu et al. (2018) demonstrated that the disruption of starch synthesis resulted in a significant increase in FA synthesis via post-translational regulation of the plastidial acetyl-coenzyme A carboxylase and a concurrent increase in the synthesis and turnover of membrane lipids and triacylglycerol.

**Figure 8.**
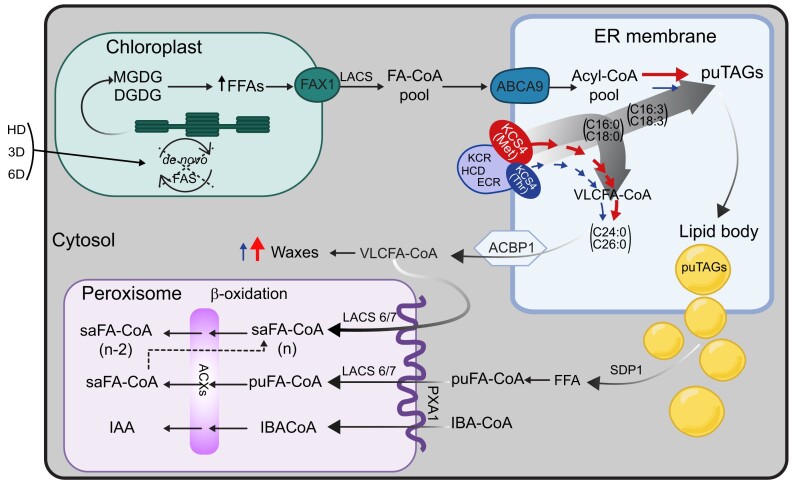
Model summarizing the role of KCS4 on FA fate in Arabidopsis under carbon starvation. The lack of de novo FA synthesis under heat and darkness (HD) or extended darkness (3D, 6D) induces the degradation of galactolipids (MGDGs and DGDGs) from the chloroplasts’ thylakoids. FFAs are then exported to the ER (FA-CoA pool), where KCS4 is localized and acts as a branch point in the fate of FAs, channeling saturated FAs to VLCFA elongation and tipping the balance to a higher polyunsaturated-to-saturated-FA ratio for puTAG accumulation (degrade grey arrows). Two *KCS4* alleles have differential capacity to allocate saturated FAs into the VLCFA pathway, with a major -more efficient- allele, *KCS4*(Met), and a minor -weaker allele, *KCS4*(Thr), exemplified here in different sizes. *KCS4*(Met) accessions efficiently channel the saturated FA from the Acyl-CoA pool to cuticular waxes, as they present higher accumulation of waxes than *KCS4*(Thr) accessions under stress. In addition, polyunsaturated TAG (puTAGs) levels are, therefore, higher in *KCS4*(Met) accessions compared to *KCS4*(Thr) accessions, as the result of a rise in the ratio of polyunsaturated-to-saturated FAs going to TAG formation. VLCFA-CoA produced by the elongase complex (here exemplified by KCS4, KCR, HCD, ECR) go to increase the cuticular waxes under HD and/or directly serve as a source of carbon undergoing degradation in the peroxisome; whereas puTAGs accumulate into lipid bodies first. FFAs are then released from lipid bodies by the action of lipases (SDP1) and further imported to the peroxisome to undergo β-oxidation. Acronyms: ABCA9 (ATP-BINDING CASSETTE A9; AT5G61730), ACBP-1 (Acyl-CoA-binding protein 1; AT5G53470); ACX (ACYL-COA OXIDASE); CoA (coenzyme A); DGDG (digalactosyldiacylglycerol); ECR (ENOYL-COA REDUCTASE; AT3G55360); FAs (fatty acids); FAS (fatty acid synthesis); FAX1 (FATTY ACID EXPORT1; AT3G57280); FFAs (free fatty acids); HCD (β-HYDROXYACYL-COA DEHYDRATASE); IAA (indol-3-acetic-acid), IBA (indol-3-butyric-acid), KCR (β-KETOACYL REDUCTASE); KCS4(Met/Thr) (3-KETOACYL-COA SYNTHASE4); LACS (LONG-CHAIN ACYL-COA SYNTHETASE); SDP1 (SUGAR DEPENDENT1; AT5G04040); MGDG (monogalactosyldiacylglycerol); PXA1 (PEROXISOMAL ABC-TRANSPORTER 1; AT4G39850); pu (polyunsaturated), sa (saturated), TAGs (triacylglycerols); VLCFA (very long chain fatty acid).

To summarize our findings, we present a model of lipid remodeling under heat and dark stress in Arabidopsis (Fig. 8). In the proposed model, darkness induces the predominant response within the combined stress, blocking de novo FA synthesis (de novo FAS) and producing considerable membrane-lipid remodeling. We observed decreased levels of MGDGs and DGDGs across accessions, as previously shown (Burgos et al. 2011). Under carbon starvation, FAs, mostly 16:3 and 18:3, are released from galactolipids by lipases (Higashi et al. 2015; Fan et al. 2017). FAs are then exported to the ER, where KCS4 is localized and acts as a branch point in the fate of FAs, directing the saturated ones to the VLCFA. Two *KCS4* alleles have differential capacities to allocate saturated FAs into the VLCFA pathway, with a major, more efficient allele, *KCS4*(Met), and a minor, weaker-allele, *KCS4*(Thr). *KCS4*(Met) accessions efficiently channel the saturated FA from the Acyl-CoA pool to cuticular waxes, as they present a higher accumulation of waxes than *KCS4*(Thr) accessions under stress (Fig. 4). Polyunsaturated TAG levels are, therefore, higher in *KCS4*(Met) accessions compared to *KCS4*(Thr) accessions, as the result of a rise in the ratio of polyunsaturated-to-saturated FAs that proceeds to TAG formation (Fig. 4). This effect of *KCS4*(Met) on TAG levels is only observed under stress conditions, when there is an increased amount of free-PUFA coming from membrane lipids, and when it is necessary to sequestered them into TAGs, to protect the cell from the FFA-toxicity induced damage (Kao et al. 2018).

Our work demonstrates that KCS4 is a key enzyme involved in the regulation of puTAG levels under heat and darkness and under extended darkness alone, adding complexity to lipid metabolism regulation under carbon starvation. The enzymatic function of KCS4 in the synthesis of VLCFA affects the accumulation of the unsaturated Acyl-CoA pool (e.g. 16:3 and 18:3) and the sequestration of unsaturated Acyl-CoA into puTAGs. Despite being a member of a 21-gene family with overlapping function in VLCFA synthesis and some redundancy in spatial and temporal regulation of expression, these results single out KCS4 as functioning in fine-tuning lipid regulation, and show that polymorphisms in KCS4 might have helped plants adapt to different geographic regions.

In conclusion, our study demonstrates that lipidomic GWAS performed under stress conditions is a powerful and unbiased tool for shedding light on the genetic control of lipid metabolism. We identified a QTL to which lipids are mapped in an environment-specific fashion, thus illustrating the plasticity of the genome in the regulation of lipid metabolism in changing environments. This approach fleshed out the fundamental role that KCS4 has in FA channeling under stress and proved to be powerful in unraveling the genetic regulation of complex phenotypes.

## Materials and methods

### Plant material

The GWA mapping population comprised a total of 330 *A. thaliana* natural accessions belonging to the HapMap panel (Horton et al. 2012). This panel was selected because the accessions maximize diversity and minimize redundancy and close family relatedness (Baxter et al. 2010).

### GWAS experimental design

Accessions or mutant lines were grown in soil (potting compost) in short days (8 h light) for 4 wk, then transferred to long days (16 h light) for an additional period of 2 wk. Control plants were harvested immediately and stressed plants were subjected to the treatment and harvested right after it.

In control conditions (for heat/dark experiment: CHD, for dark experiment: CD), plants were grown on long days (16 h light, 8 h dark), temperature was kept at 21/16 °C (day and night, respectively), light intensity at 150 *μ*E m^−2^ s^−1^, and humidity at 75%. In the heat and dark experiment (HD), plants were grown like the plants in CHD for 6 wk, then subjected to dark and 32 °C continuously for 24 h, and then harvested. In the dark treatment, plants were grown like the plants in CD for 6 wk and then transferred to continuous darkness either for 72 h (3D) or 144 h (6D). Plants from HD, 3D, and 6D were harvested 24, 72, and 144 h after the beginning of the treatment, respectively. Between 3 and 6 plants from each accession/line were collected for each condition tested and extracted for lipid extraction (see below). We followed a similar experimental design to that described in our recently published study (Zhu et al. 2022)

### Targeted lipid profiling by LC-MS and data acquisition

Lipid extraction was done, and measurements from Arabidopsis leaves (100 mg for each sample) were performed as described previously (Hummel et al. 2011). In brief, dried organic phase was measured using Waters Acquity ultra-performance liquid chromatography system (Waters, http://www.waters.com) coupled with Fourier transform mass spectrometry (UPLC-FT-MS) in positive ionization mode. Analysis and processing of raw MS data were done with REFINER MS 10.0 (GeneData, http://www.genedata.com). Workflow included peak detection, retention time alignment, and removal of chemical noise and isotopic peaks from the MS data. Obtained mass features characterized by specific peak ID, retention time, *m*/*z* values, and intensity were further processed using custom scripts in R (R Core Team 2019). Clusters with means of signal intensities lower than 50,000 were removed, and only peaks present in at least 70% of the samples were kept for further analysis. Peak intensities were normalized by the day of measurement and median and subsequently log_2_ transformed. After that, obtained molecular features were queried against a lipid database for annotation. The in-house database used includes 219 lipid species of the following classes: diacylglycerols (DAGs), DGDGs, MGDGs, phosphatidylcholines, phosphatidylethanolamines, and TAGs. This was carried out by comparing their retention times and exact masses against those of the reference compounds, allowing maximal deviations of 0.1 min and 10 ppm. Identified lipids were confirmed by manual verification of the chromatograms using XCalibur (Version 3.0, Thermo-Fisher, Bremen, Germany) (Alseekh et al. 2021).

### General statistical and multivariate analysis of lipidomic data

To calculate the correlation between lipids, the Pearson correlation was used. The correlation plots and heatmaps were created using the package pheatmap (Raivo Kolde 2019). The lipids were clustered using complete-linkage clustering. PCA was obtained from the correlation matrix of lipidomic data (CHD and HD together) using the pcaMethod (Stacklies et al. 2007) package.

To detect the fold change in lipid feature levels across all accessions for each lipid feature, fold change was calculated by dividing the average of the 5 highest intensities measured in a given accession by the average of the 5 lowest intensities, (i) for all annotated lipids (*N*_CHD_ = 98, *N*_HD_ = 109), (ii) only for TAGs (*N*_CHD_ = 30, *N*_HD_ = 41), and (iii) for lipid classes other than TAGs (*N*_CHD_ = 68, *N*_HD_ = 68).

### GWAS analysis

Data acquisition for GWAS, mapping, and locus identification were performed as described previously (Wu et al. 2016; Fusari et al. 2017). GWAS was performed using a compressed mixed linear model (MLM) implemented in the GAPIT (Lipka et al. 2012) package. The 109 (HD), 98 (CHD), 80 (3D), 109 (6D), and 122 (CD) lipid features were used as phenotypes (Supplemental Data Set S1), whereas a set of 214,051 SNPs was used as genotypic data (250 K SNPchip). A total of 3 principal components were included in the MLM to account for population structure, and the SNP fraction considered to perform PCA was set to 0.1 to avoid excessive computations. Kinship matrix and other parameters were set to default values.

Additionally, we performed GWAS using a full genome sequence from a section of Chromosome 1. Shortly, sequences for 80 kb around *KCS4* (AT1G19440, TAIR10 position: 6689119–6769118) were obtained from the Arabidopsis 1001 Genomes project (http://signal.salk.edu/atg1001/3.0/gebrowser.php, 162 accessions). The alignment was performed using the default routine implemented in MAFFT (Katoh et al. 2019) with a final manual inspection to discard alignment problems due to big gaps or missing data (Supplemental File S1). For 9 accessions, 2 sequences were available. The sequences included further in the analysis were selected based on the agreement of alleles with the SNPchip data and/or on the percentage of nonmissing data (≥90%). Final SNP matrix, obtained by filtering for minor allele frequency ≥ 5%, contained 1,213 SNPs and indels (Supplemental Data Set S4). This matrix was used as the genotypic data. Population structure and cryptic relationships among accessions were taken into account by including principal components as cofactors and a kinship matrix obtained with SNPs in the 250 K SNPchip for the subset of 162 accessions (Supplemental Data Set S4). Lipid features collected previously (HD and 3D) Supplemental Data Set S1) were used as phenotypes. The 3 data sets (genotype, population structure, and phenotypes) matched for 149 accessions in the HD experiment and for 134 accessions in the 3D experiment. MLM was implemented using TASSEL software with the default settings for compressed MLM (Bradbury et al. 2007).

### SNP filtering, QTL identification, and candidate gene selection

For results obtained using GAPIT package, only SNPs having a *P*-value < 1/*N* (*N* = 214,051) were retained to correct for multiple comparisons. These SNPs were assigned to the same QTL if the genomic distance between them was ≤10 kb. All genes in a given QTL were taken as putative candidates. Further candidate gene selection for validation was done based on functional annotation or previous knowledge.

For results obtained using Tassel software, SNPs were regarded as significantly associated with lipid traits after setting Bonferroni threshold (*α* = 0.05/*N*, *N* = 1,213 polymorphism tested).

### LD, nucleotide diversity, and haplotype analysis

LD was computed as the coefficient of correlation between pair of sites (*r*^2^) using the LDheatmap package (Shin et al. 2006), either with the SNPchip (for 1,300 and 314 accessions) or with the full sequence data (for 149 accessions). For deviations from neutrality, Tajima's *D* statistic (Tajima 1989) was computed as described previously (Fusari et al. 2017).

Haplotypes for *KCS4* were obtained by using SNPchip data considering 1,000 bp upstream of the ATG, the coding region and 1,000 bp downstream of the stop codon (SNPs = m11502, m11503, m11504, m11505). Accessions belonging to the same haplotype were grouped and the haplotype median was obtained for each lipid feature. To cluster haplotypes, a combination of allele sharing distance and Ward's minimum variance was used, as described before (Gao and Starmer 2007; Qiu et al. 2015). One-way ANOVA followed by Bonferroni correction for multiple comparisons was performed to test differences between haplotypes (*P* < 0.05).

### Mutant line selection and genotyping

For Arabidopsis CRISPR-Cas9 lines, the binary vector containing guide RNAs (gRNAs) and the Cas9 nuclease were generated based on a classic cloning procedure described previously (Schumacher et al. 2017). A pair of gRNAs was used to target the N-terminal coding sequence of *KCS4* using the spacer sequences indicated in Supplemental Data Set S7. As background, wild-type accessions carrying *KCS4*(Met): Pro-0, Petergof, Per-1, Col-0; and *KCS4*(Thr): Si-0, Bu-8, Sh-0 were chosen based on TAG levels phenotyped in HD. Mutations were confirmed by PCR and sequencing of *KCS4* using primers listed in Supplemental Data Set S7. Sequence contigs and alignment were done using Bioedit (Hall 1999).

For heterologous expression in yeast and transient expression in *N. benthamiana, KCS4* (AT1G19440) and *KCS9* (AT2G16280) open reading frame was amplified from Arabidopsis flower cDNA using primers listed in Supplemental Data Set S7. The corresponding PCR fragments were inserted into the pDONR 221 ENTRY vector using the GATEWAY recombinational cloning technology (Invitrogen, Darmstadt, Germany) and subsequently transferred into pK7YWG2, pK7WG2D, and pvtLEU DESTINATION vectors (Karimi et al. 2002; Dittrich-Domergue et al. 2014) by using LR clonase II. All transient heterologous expressions in *N. benthamiana* were made using the *Agrobacterium tumefaciens* strain GV3101 and 5-week-old plants cultivated in controlled conditions (16-h light photoperiod, 25 °C) (Perraki et al. 2012). *N. benthamiana* plants were agroinfiltrated on the abaxial side of the leaves and analyzed 2 and 5 d after agroinfiltration for subcellular localization experiments and for overexpression, respectively.

For yeast experiments, *Saccharomyces cerevisiae* strains INVSc1 (MATa *his3*Δ*1 leu2 trp1-289 ura3-52*) and Δ*elo3* (MATa *his3*Δ*1 leu2 trp1-289 ura3-52 elo3*Δ) were used to express *KCS4* wild-type and allelic mutants and *KCS9*.

### RT-qPCR

*RGS1-HXK1 INTERACTING PROTEIN1/RHIP1* (AT4G26410) was used as a reference gene for *KCS4* transcript analysis in CHD and HD for the complete GWAS population (Czechowski et al. 2005). *GAPDH3* (AT1G13440) was used as a reference gene for starvation markers and *KCS* transcript analysis. All primers used are listed in Supplemental Data Set S7.

RT-qPCR was performed on 3 individual plants per ecotype. Each sample was tested in 3 technical replicates. Averaged values for each ecotype were pooled together according to the allele carried. For CHD *n* = 193 for the C allele and *n* = 94 for the T allele. For HD *n* = 163 for the C allele and *n* = 75 for the T allele. RT-qPCR was performed on an ABI Prism 7900 HT real-time PCR system (Applied Biosystems/Life Technologies, Darmstadt, Germany) in 384-well PCR plates with a total reaction volume of 5 *μ*L (2 *μ*L forward and reverse primer mixture, 0.5 *μ*M each), 0.5 *μ*L cDNA and 2.5 *μ*L Power SYBR Green-PCR Master Mix (Applied Biosystems/Life Technologies) using the following cycling program: 50 °C for 2 min, 95 °C for 5 min, 40 cycles of 95 °C for 15 s, and 60 °C for 60 s, followed by a denaturation step of 95 °C for 15 s and 60 °C for 15 s, followed by a continuous temperature increase (0.3 °C s^−1^) to 95 °C (for 15 s). Steps 5, 6, and 7 were introduced to record a dissociation or melting curve for each product in order to detect nonspeciﬁc ampliﬁcation. Expression values were calculated by 2^(−ΔΔCt)^ using either *ACTIN 2* (AT3G18780) or *GAPDH3* (AT1G13440) as reference genes. Errors are given as the lower (2^(−ΔΔCt−Sd)^) and upper (2^(−ΔΔCt^^+^^Sd)^) limit of the standard deviation around the mean.

### EMSA

EMSA reactions were performed as previously described (Thirumalaikumar et al. 2018). Briefly, HSFA2-GST proteins were prepared as stated before (Puranik et al. 2011). *HSFA2* coding sequence was PCR-amplified from Arabidopsis heat-stressed cDNA using primers listed in Supplemental Data Set S7. PCR products were recombined in pDEST24 using the GATEWAY cloning system (Invitrogen). Recombinant vectors were transformed into *Escherichia coli* Star (DE3) pRARE generated by transforming the pRARE plasmid isolated from Rosetta (DE3). 5′ labeled with fluorescent dye DY682 and unlabeled *KCS4* promoter regions spanning the SNP position m11502 (C or T allele) were purchased from Eurofins MWG operon. DNA EMSA reactions were performed using Odyssey Infrared EMSA kit (Li-COR, Bad Homburg, Germany). Competitor was added at 200× the amount of probe.

### Fatty acid composition and content analysis for *N. benthamiana and yeast*

For fatty acid methyl esters (FAMEs) analysis in *N. benthamiana*, 3 agroinfiltrated leaf disks were transferred to tubes containing 1 mL of 0.5 M sulfuric acid in methanol containing 2% (v/v) dimethoxypropane and 20 *μ*g of heptadecanoic acid (C17:0) as an internal standard. Transmethylation was performed at 85 °C for 3 h. For FAMEs analysis in yeast, yeasts were grown in 5 mL of appropriate liquid minimal medium for 1 wk at 30 °C, then pelleted and washed in 2 mL of 2.5% (w/v) NaCl. Yeast cell pellets were resuspended in 1 mL of 0.5 M sulfuric acid in methanol containing 2% (v/v) dimethoxypropane and 20 *μ*g of heptadecanoic acid (C17:0) as internal standard and transmethylation was performed at 85 °C for 1 h. After cooling, 2 mL of 2.5% (w/v) NaCl was added, and fatty acyl chains were extracted in 300 *µ*L of hexane. Samples were analyzed by GC-FID and GC-MS as previously described (Domergue et al. 2010).

### Cuticular wax analysis

Epicuticular waxes were extracted from leaves from 2 to 4 different plants for each allele (*KCS4*[Met]/*KCS4*[Thr]) by immersing tissues for 30 s in chloroform containing docosane (C22 alkane) as an internal standard. Extracts were derivatized and analyzed as previously described (Bourdenx et al. 2011).

### Confocal microscopy

Live imaging was performed using a Leica SP5 confocal laser scanning microscopy system (Leica, Wetzlar, Germany) equipped with Argon, DPSS, He–Ne lasers, hybrid detectors, and 63× oil-immersion objective. Two days post agroinfiltration, *N. benthamiana* leaf samples were gently transferred between a glass slide and a cover slip in a drop of water. The plasmid ER-gk CD3-955 was used as a fluorescent ER marker (Nelson et al. 2007). YFP and GFP fluorescence were observed using excitation wavelength of 488 nm and their fluorescence emission was collected at 490 to 540 nm.

### Biomass determination

Plants were grown and treated as described for lipid measurements. Plants from wild-type accessions carrying *KCS4*(Met) and *KCS4*(Thr) as well as allelic mutants *kcs4*(Met) and *kcs4*(Thr) were grown in CHD or treated for 24 h with HD and harvested right after (CHD and HD at the same time). Biomass measurement was performed on complete rosettes for each line. Results from accessions or mutants were then pooled according to the allele carried (*n* = 3 ± Sd).

### Geographic and phylogenetic signal of the lipidome

The neighbor-joining tree was constructed by BioNJ algorithm (Gascuel 1997), using J-C distance in SeaView 4.4.3 software (Gouy et al. 2010). Phylogenetic signal was computed using *K* statistics and as a background a Brownian motion model of trait evolution (Blomberg et al. 2003) in the “picante” R package (Kembel et al. 2010). Significance of the phylogenetic signal was estimated by comparison of observed variance patterns to a null model obtained by 9,999 random permutations of the accession labels across the phylogeny. The altitude at the sites was estimated using the geomapdata database (Lees 2000) based on the latitude and longitude coordinates. The climate conditions have been downloaded from the WorldClim database (www.worldclim.org, Fick and Hijmans 2017). The lipidomic data were regressed against the geodesic distance matrix using nonparametric multivariate variance analysis (Anderson 2001). The relationship between lipid levels and the climate parameters was analyzed using regularized regression model (Friedman et al. 2010). The regularization parameter alpha and penalty parameters lambda were estimated using 10-fold cross-validation within the training set. In result, the Elastic Net regularization with parameter *α* = 0.5 was selected as optimal and applied for all regression models and the “λmin + 1Se” rule was applied to choose the penalty parameter for each model separately. Prediction power was estimated in the Monte Carlo cross-validation test with 100 samples with 57% and 25% of samples taken as a training and test set, respectively. Kruskal–Wallis and Wilcoxon rank sum tests have been performed using the R stats package.

### Statistics

All statistical tests (ANOVA and *t*-test results showing variables, parameters, degrees of freedom, and tests) are included in Supplemental Data Set S8.

### Accession numbers

AT1G01120: *3-KETOACYL-COENZYME A SYNTHASE1* (*KCS1*); AT1G04220: *3-KETOACYL-COENZYME A SYNTHASE2* (*KCS2*); AT1G06250: *LIPASE CLASS 3*; AT1G06290: *ACYL-COA OXIDASE3* (*ACX3*); AT1G06310: *ACYL-COA OXIDASE6* (*ACX6*); AT1G06350: *FATTY ACID DESATURASE* (*CER17*); AT1G063609: *FATTY ACID DESATURASE* (*ADS4.2*); AT1G13440: *GLYCERALDEHYDE-3-PHOSPHATE DEHYDROGENASE* (*GAPDH3*); AT1G19440: 3-KETOACYL-COENZYME A SYNTHASE4 (KCS4); AT1G25450: *3-KETOACYL-COENZYME A SYNTHASE5* (KCS5); AT1G68530: *3-KETOACYL-COENZYME A SYNTHASE6* (KCS6); AT2G15090: *3-KETOACYL-COENZYME A SYNTHASE8* (*KCS8*); AT2G16280: *3-KETOACYL-COENZYME A SYNTHASE4* (*KCS9*); AT2G26150: *HEAT SHOCK TRANSCRIPTION FACTOR A2* (*HSFA2*), AT2G28630: *3-KETOACYL-COENZYME A SYNTHASE12* (*KCS12*); AT3G12120: *FATTY ACID DESATURASE2* (*FAD2*); AT3G18780: *ACTIN 2*; AT4G26410: *RGS1-HXK1 INTERACTING PROTEIN1/RHIP1*; AT4G34250: *3-KETOACYL-COENZYME A SYNTHASE16* (*KCS16*); AT4G34520: *3-KETOACYL-COENZYME A SYNTHASE18* (*KCS18*); AT4G39850: *PEROXISOMAL ABC-TRANSPORTER1* (*PXA1*); AT5G04040: *SUGAR DEPENDENT1* (*SDP1*); AT5G20060: *LYSOPHOSPHOLIPASE*; AT5G43760: *3-KETOACYL-COENZYME A SYNTHASE20* (*KCS20*); AT5G49070: *3-KETOACYL-COENZYME A SYNTHASE21* (*KCS21*).

## Supplementary Material

koad059_Supplementary_Data

## Data Availability

The data used to support the results of this study are provided within the article.

## Supplemental data

The following materials are available in the online version of this article.

**Supplemental Figure S1**. Phenotypic analyses for the Arabidopsis GWAS panel under control (CHD) and stress (HD) conditions.

**Supplemental Figure S2**. Results from GWAS analysis on lipidomic data from plants grown under extended darkness (3D, 6D) conditions.

**Supplemental Figure S3**. Quantification of starvation markers in *KCS4* wild-type accessions and allelic mutants.

**Supplemental Figure S4**. KCS4 enzymatic activity.

**Supplemental Figure S5**. Quantification of *KCS4*, *KCS5*, *KCS9*, and *KCS12* transcripts in wild-type *KCS4*(Met)/*KCS4*(Thr) accessions and *kcs4*(Met)/*kcs4*(Thr) allelic mutants.

**Supplemental Figure S6**. Phylogenetic signal of the lipidome.

**Supplemental Figure S7**. Prediction of climate parameters from lipidomic profiles.

**Supplemental Data Set S1**. Phenotypic data used for GWAS.

**Supplemental Data Set S2**. GWAS results.

**Supplemental Data Set S3**. Candidate genes and QTL colocalization.

**Supplemental Data Set S4**. Data used for GWAS using complete sequenced information from 162 Arabidopsis ecotypes.

**Supplemental Data Set S5**. GWAS result using complete sequenced information from 162 Arabidopsis ecotypes.

**Supplemental Data Set S6**. Metabolomic analysis of *KCS4* wild-type alleles and *kcs4* allelic mutants.

**Supplemental Data Set S7**. Primers and probes used in this study.

**Supplemental Data Set S8**. Statistics **Supplemental File S1**. Alignment of *KCS4* QTL using complete sequenced information from 162 Arabidopsis ecotypes.

**Supplemental File S2**. Alignment of *KCS4* genomic region from wild-type Arabidopsis ecotypes and CRISPR-Cas allelic mutants used in this study.
