# Supplementary material for: Hello darkness, my old friend: 3-KETOACYL-COENZYME A SYNTHASE4 is a branch point in the regulation of triacylglycerol synthesis in Arabidopsis thaliana
Source: Plant Cell. 2023 Mar 3;35(6):1984–2005. doi: 10.1093/plcell/koad059 (PMC10226560; doi:10.1093/plcell/koad059)
Supplement: koad059_Supplementary_Data [file koad059_supplementary_data.zip › TPC2022LSB01061DR1_Supplemental.pdf]

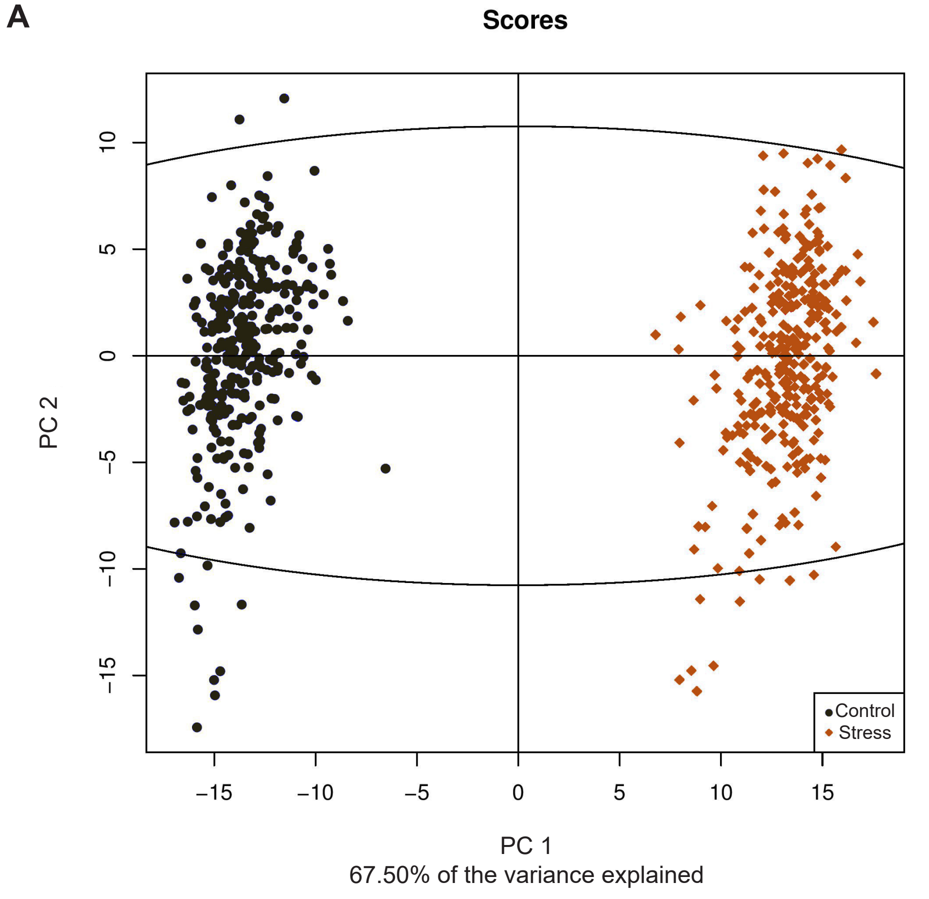

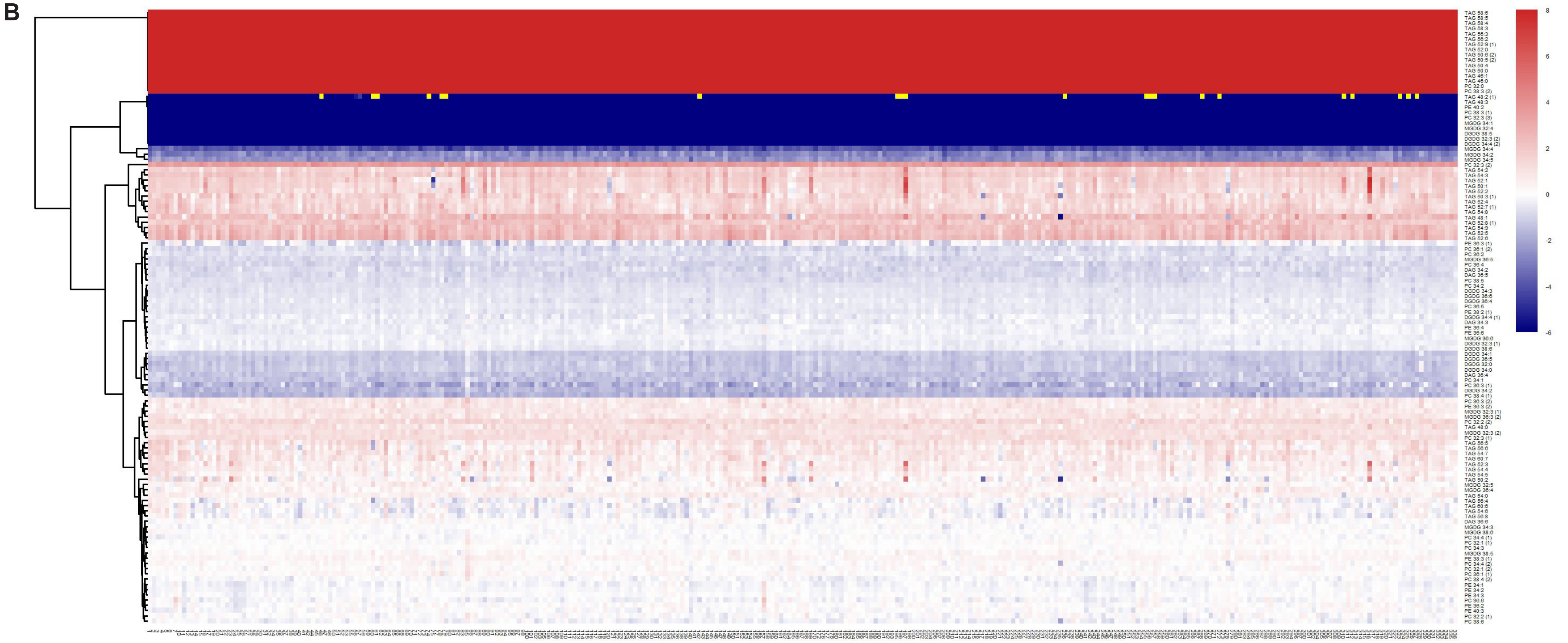

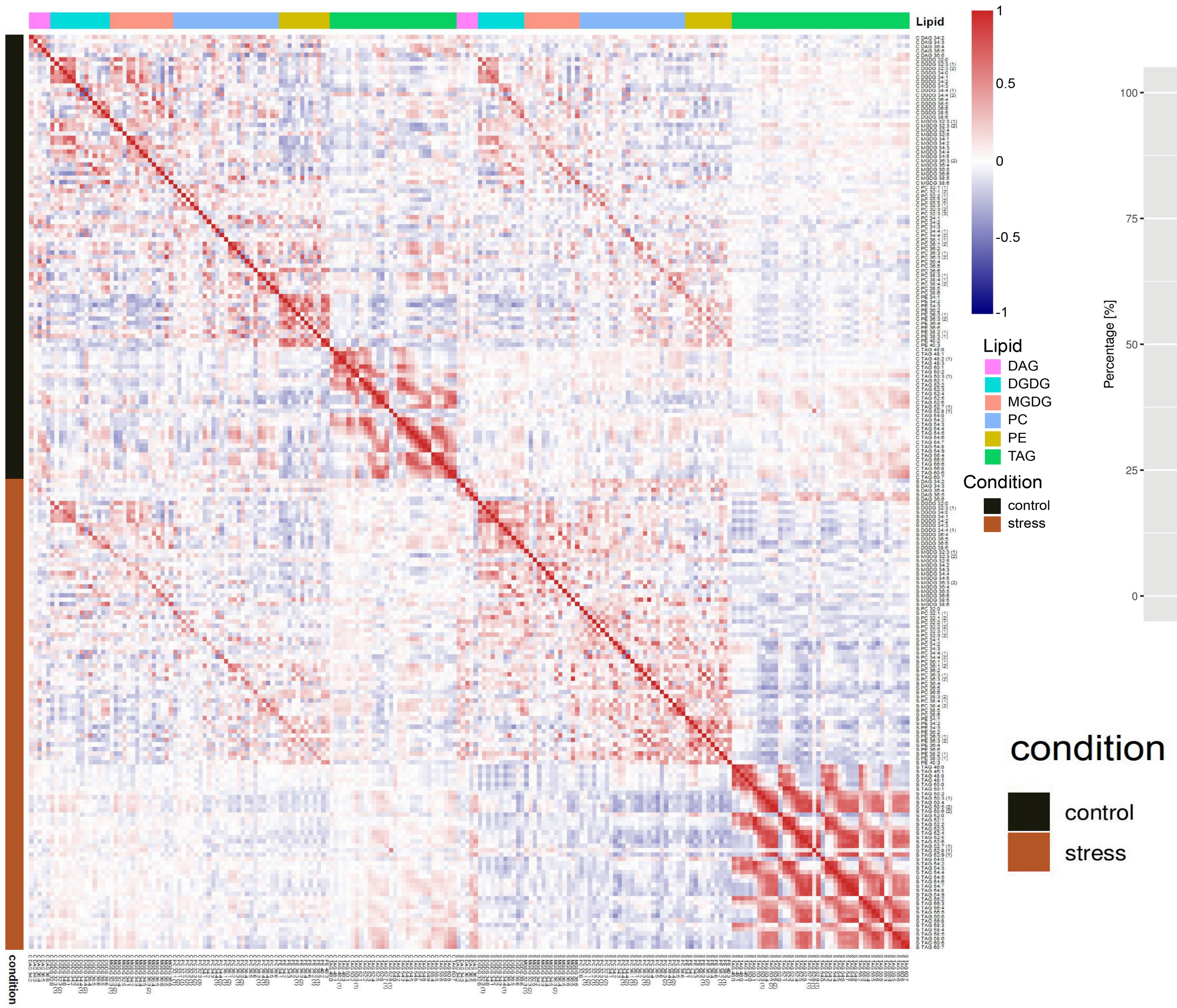

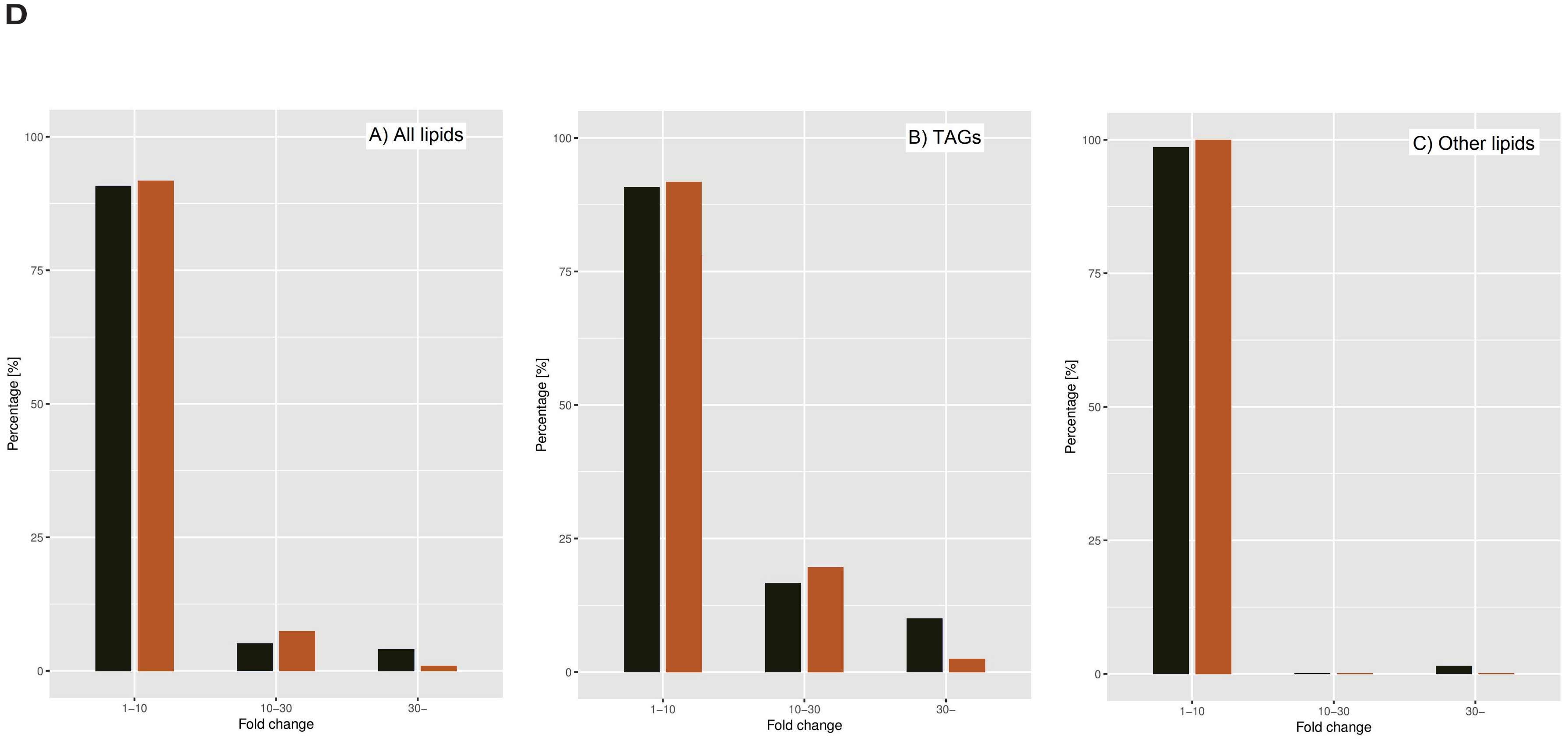

Supplemental Figure S1. Phenotypic analyses for the Arabidopsis GWAS panel under control and stress conditions

A. Principal component analysis (PCA) of lipid data for control condition (CHD, dark grey circles) and heat and dark condition (HD, orange diamonds). Each dot represents a single accession. PC1 and PC2 explain together 67.5% of the variance observed in the phenotypic data. **B.** Heatmap of differential accumulation of lipid levels in stress (HD) and control (CHD) conditions. Lipids are clustered by lipid class. For each accession, the lipid level in HD was subtracted by the lipid level in CHD. Positive values (red) denote higher levels of lipid in HD condition, negative values (blue) denote higher level of lipid in CHD condition (scale= $\log_2$ of lipid intensity). **C.** Correlation pattern among lipid levels in both conditions: CHD (dark grey rectangle) and HD (orange rectangle). Pairwise Pearson correlations (r^2) were calculated between each lipid across all accessions. Lipids are highlighted with colors according to lipid classes (references included in the figure). **D.** Distribution of fold change of lipid–feature levels across all accessions. For each lipid feature, fold change was calculated by dividing the average of the five highest intensities measured in a given 5 accession by the average of the five lowest intensities. Y-axes is expressed in percentage of (A) all metabolite features (N=109 in stress, N=98 in CHD), (B) only TAG features (N=41 in stress, N= 30 in CHD) and (C) other lipid features (N=68 in stress and N=68 in CHD). This figure supports Figure 1.

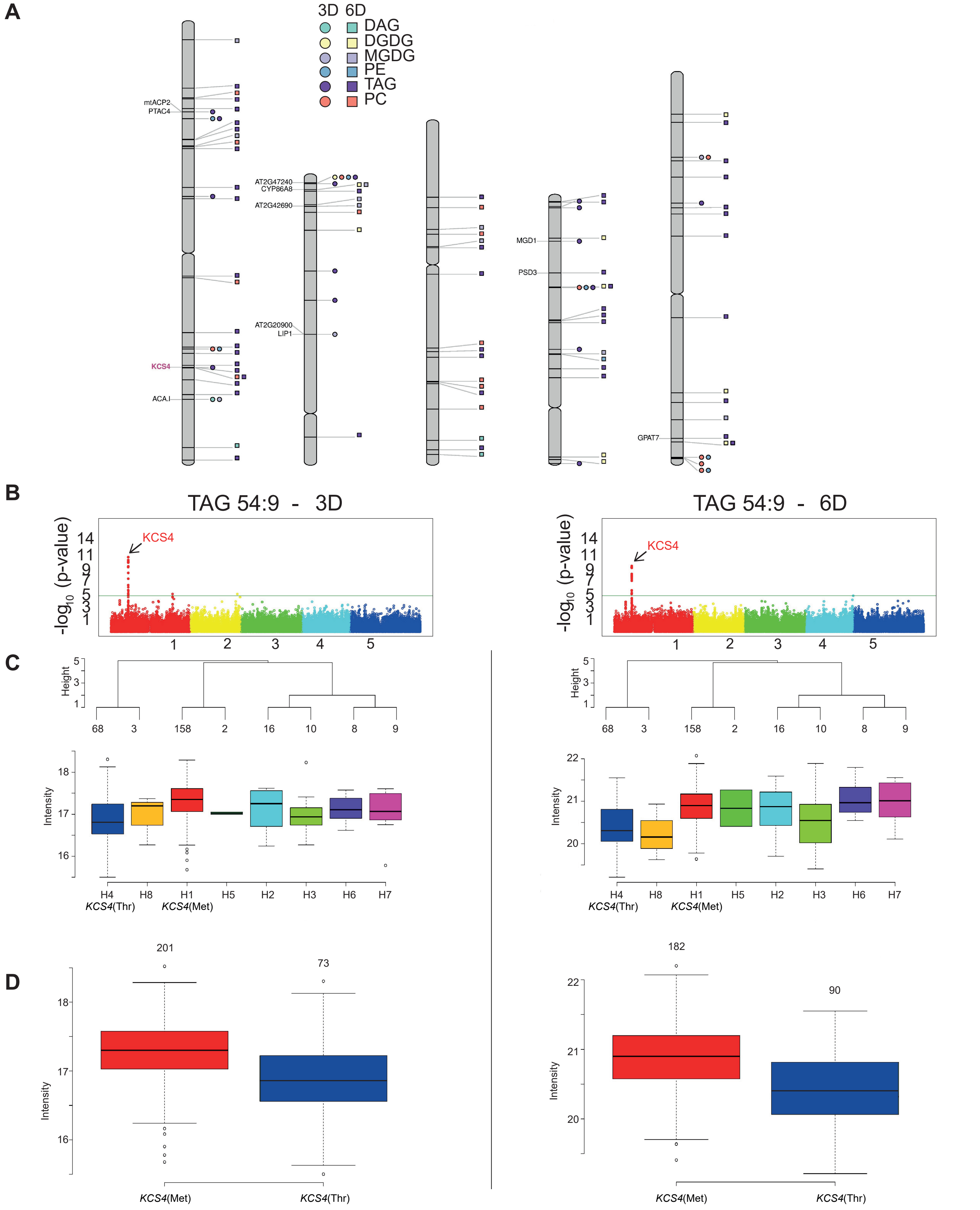

Supplemental Figure S2. Results from GWAS analysis on lipidomic data from plants grown under extended darkness (3D, 6D) conditions.

A. Chromosome scheme and Quantitative Trait Loci (QTL) identified in the two experimental conditions (3D = circles, 6D = squares), for different lipid classes: phosphatidylethanolamine (PE), phosphatidylcholine (lecithin) (PC), monogalactosyldiacylglycerol (MGDG), digalactosyldiacylglycerol (DGDG), diacylglycerol (DAG) and triacylglycerol (TAG). Color/shape references are included in the figure. Gene IDs are included only for QTL co-localizing for two or more lipid species with LOD score > 5.3. **B.** Manhattan plots obtained for TAG 54:9 in 3D (left) and in 6D (right). **C.** Average trait value (intensity of TAG 54:9, log_2 scale) for the different *KCS4* haplotypes using SNPs m11502, m11503, m11504 and m11505. **D.** TAG 54:9 average value (log_2 scale) for accessions carrying the *KCS4*(Met) and *KCS4*(Thr) alleles. **C-D.** Left panels are 3D and right panels are 6D condition. This figure supports Figure 1.

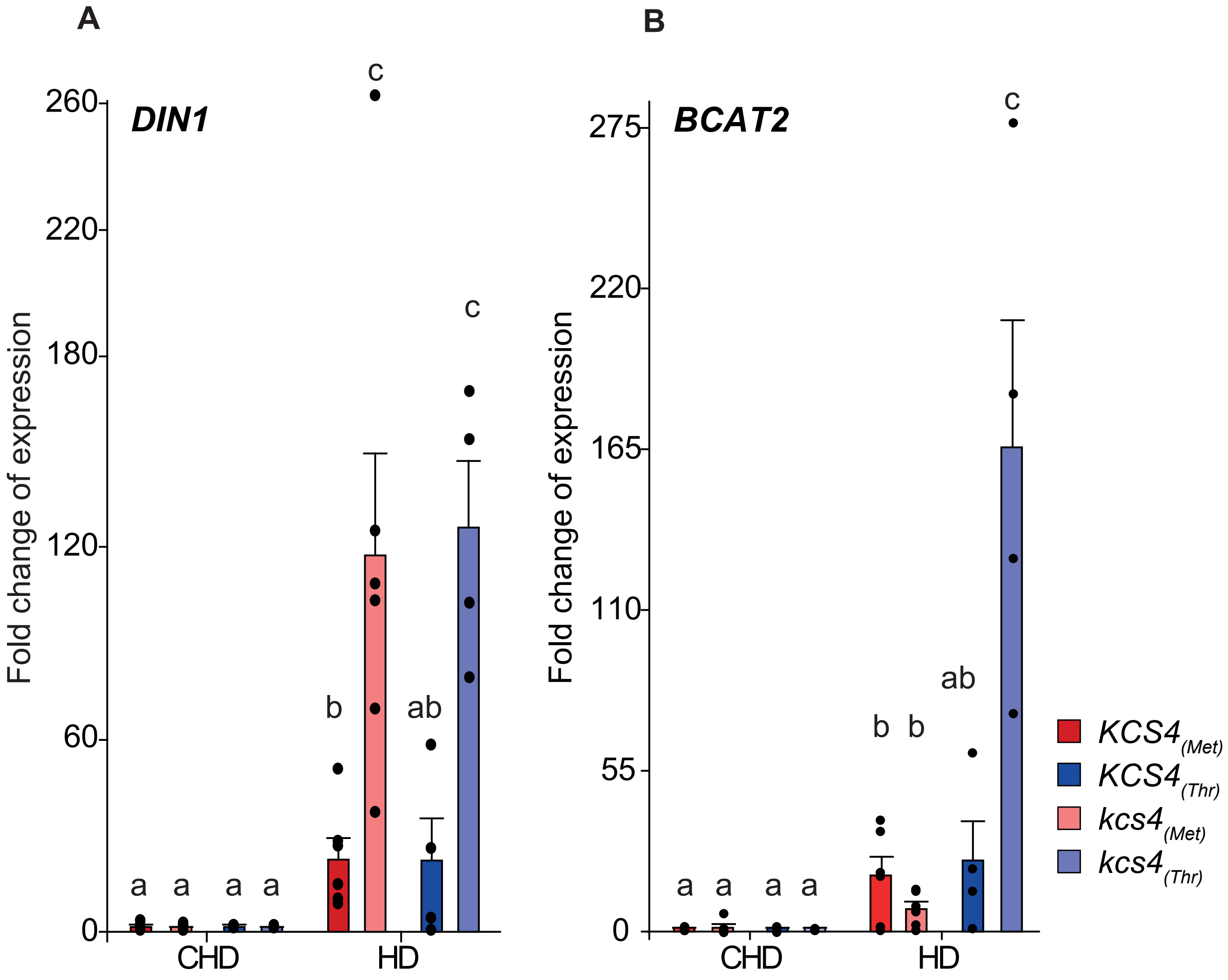

Supplemental Figure S3. Quantification of starvation markers in *KCS4* wild-type accessions and allelic mutants.
Transcripts were measured in five witld-type accessions and five allelic mutants. Three biological replicates were use for each accession, results from separate accessions were pooled according to the allele carried: *KCS4*(Met) (WT: red, mutant: pink) and *KCS4*(Thr) (WT: blue, mutant: light-blue). Plants were grown in control conditions (CHD) or subjected to heat and darkness for 24 h (HD) and harvested both group at the same time point. Fold change of expression of **(A)** *DIN1* (AT4G35770) and **(B)** *BCAT2* (AT1G10070) were obtained using the $2^{-(\Delta\Delta CT)}$ method using *GAPDH3* (AT1G13440) as housekeeping gene. Bars represent means ± SE (n= 4 to 7), individual values are plotted as black circles. Statistical significance: *ANOVA* followed by Tukey post hoc test was used to detect differences within conditions and *t*-Test was used to evaluate differences between conditions for the same allele (P < 0.05). Means with a common letter are not significantly different (P > 0.05), both tests are combined. This figure supports Figure 1.

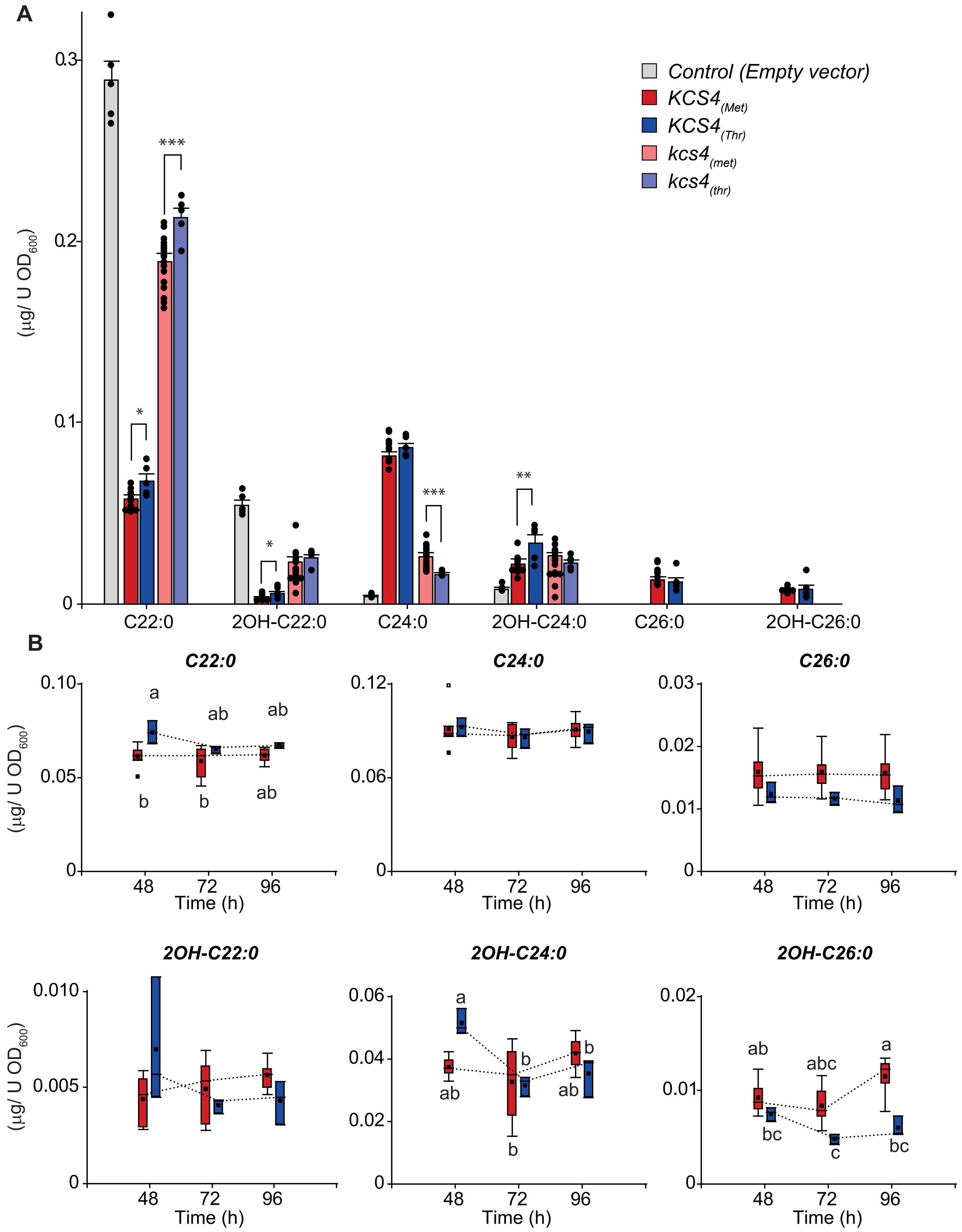

Supplemental Figure S4. KCS4 enzymatic activity.

A. Very long chain fatty acid methyl ester (VLC-FAME) profiles (22 to 26) of Δelo3 yeast mutant transformed with At*KCS4*(Met) allele (WT: red, mutant: pink) or At*KCS4*(Thr) allele (WT: blue, mutant: light-blue). Bars represent means ± SE (n=5 to 20), individual values are plotted as black circles. Significant differences between alleles were obtained with *t*-Test (* $P < 0.05$, ** $P < 0.01$, *** $P < 0.001$). **B.** VLC-FAME profiles (22 to 26) at different times of harvest (48, 72 and 96 h) for At*KCS4*(Met) and At*KCS4*(Thr) wild-type alleles (red and blue, respectivetly; n=3 to 6). Significant differences were obtained with ANOVA followed by Tukey post hoc test ($P < 0.05$). Means with a common letter are not significantly different. This figure supports Figure 5.

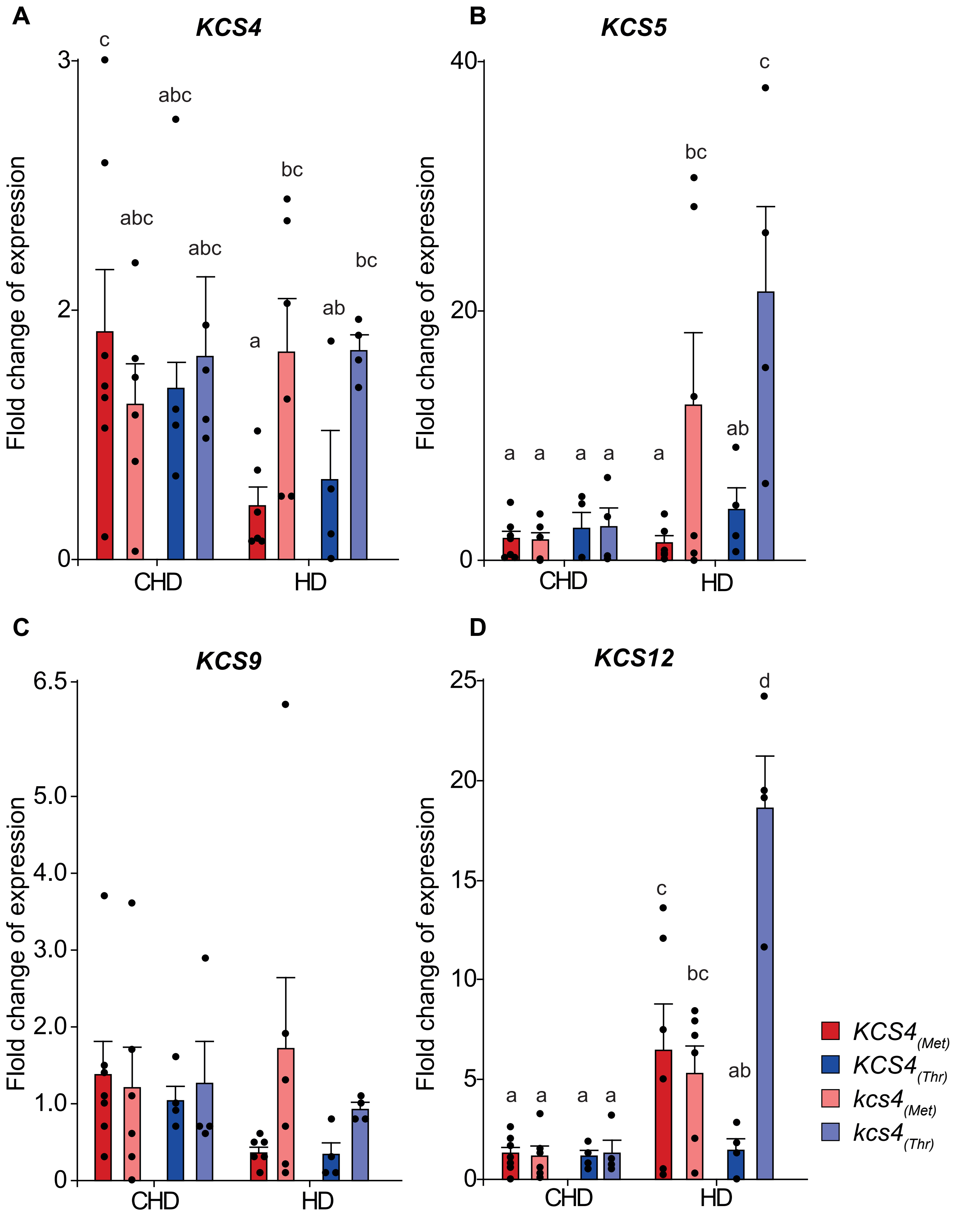

Supplemental Figure S5. Quantification of *KCS4*. *KCS5*. *KCS9* and *KCS12* transcripts in *KCS4*(Met)/*KCS4*(Thr) wild-type accessions and *kcs4*(Met)/*kcs4*(Thr) allelic mutants.
Transcripts were measured in five wild-type accessions and five allelic mutants. RNA from three different plants were extracted independently for each accession, results from separate accessions were pooled together according to the allele carried *KCS4*(Met) (WT: red, mutant: pink) and *KCS4*(Thr) (WT: blue, mutant: light-blue). Plants were grown in control conditions (CHD) or subjected to heat and darkness for 24 h (HD). Fold change of expression of **(A)** *KCS4* (AT1G19440), **(B)** *KCS5* (AT1G52450), **(C)** *KCS9* (AT2G16280) and **(D)** *KCS12* (AT2G28630) were obtained using the $2^{-(\Delta\Delta CT)}$ method using *GAPDH3* (AT1G13440) as housekeeping gene. Bars represent means ± SE (n = 4 to 7), individual values are plotted as black circles. Statistical significance: *ANOVA* followed by Fisher-LSD post hoc test was used to detect differences within and between conditions (P < 0.05). Means with a common letter are not significantly different (P > 0.05). This figure supports Figure 3.

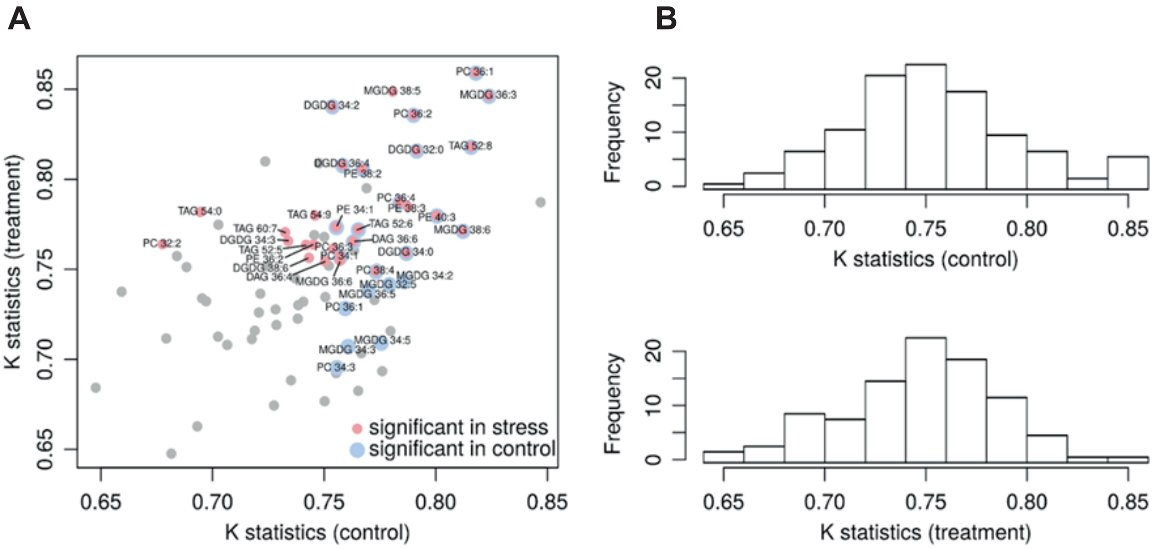

Supplemental Figure S6. Phylogenetic signal of the lipidome

A. Distribution of the phylogenetic signal calculated for all measured lipids in HD and CHD conditions. Only lipids exhibiting significant phylogenetic signal are labelled. Lipids significant in CHD and HD are marked with blue and red dots, respectively. Compounds significant in both conditions ($P < 0.05$) have overlapping red and blue markers. **B.** K parameter distribution obtained for the TAG profiles in CHD (control) and HD (treatment) condition according to (Blomberg et al., 2003). This figure supports Figure 7.

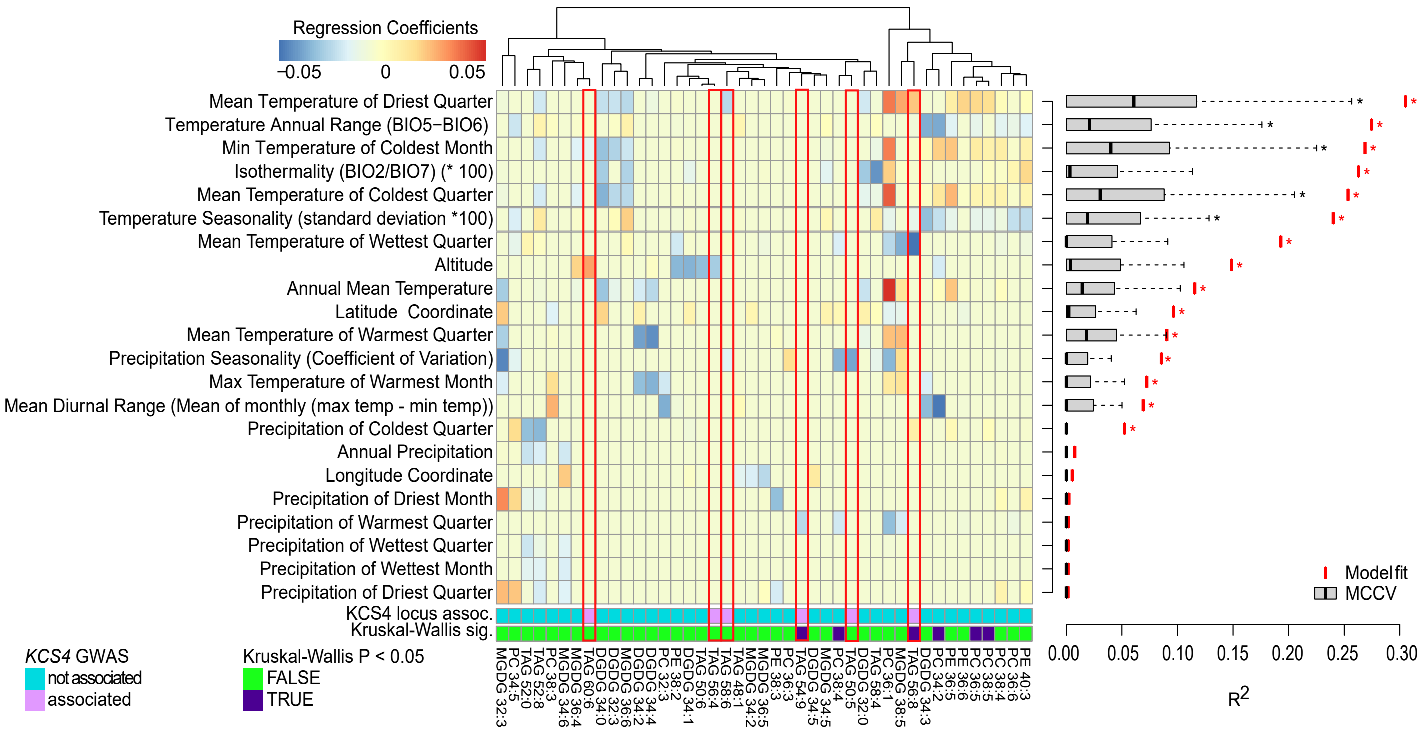

Supplemental Figure S7. Prediction of climate parameters from lipidomic profiles

Regression coefficients of selected predictive lipidomic features towards 19 climate variables and 3 geographical coordinates (latitude, longitude and altitude) of the geographical origin of analyzed accessions. Statistical evaluation of the fitted models is provided on the right-hand panel. The model fit significance (red asterisks) is estimated by the correlation coefficient between the original and model-fitted variable values. The significance of Cross-Validation test (black asterisks) represents the significance of the correlation coefficient between the predicted and real values of the test sample. The cross-validation has been performed 100 times with the 3:1 proportion of the training to test sample set size. This figure supports Figure 7.

Supplemental File 1. Alignment of *KCS4* QTL using complete sequenced information from 162 Arabidopsis ecotypes

Alignment used for the generation of the SNP matrix used in TASSEL to analyze more polymorphisms in the QTL associated with *KCS4*. Sequences were obtained from the Arabidopsis 1001 genome project (http://signal.salk.edu/atg1001/3.0/gebrowser.php, 162 accessions). TAIR10 positions of the extract are chromosome 1: 6689119 - 6769118. Alignment was performed using MAFFT (Katoh et al., 2019) and manually curated when needed.

Supplemental File 2. Alignment of *KCS4* genomic region from wild-type Arabidopsis ecotypes and CRISPR-Cas allelic mutants used in this study

KCS4 genomic region was amplified in wild-type accessions (Col-0, Petergof, Pro-0, Sh-0, Si-0, Per-1 and Bu-8) and allelic mutants *kcs4*(Met)/*kcs4*(Thr) and, and sequenced to confirm mutations. Primers used for amplification and sequencing are described in Dataset 7.
